# An Augmented-Reality fNIRS-Based Brain-Computer Interface: A Proof-of-Concept Study

**DOI:** 10.3389/fnins.2020.00346

**Published:** 2020-04-28

**Authors:** Amaia Benitez-Andonegui, Rodion Burden, Richard Benning, Rico Möckel, Michael Lührs, Bettina Sorger

**Affiliations:** ^1^Department Cognitive Neuroscience, Faculty of Psychology and Neuroscience, Maastricht Brain Imaging Center, Maastricht University, Maastricht, Netherlands; ^2^Laboratory for Cognitive Robotics and Complex Self-Organizing Systems, Department of Data Science and Knowledge Engineering, Faculty of Science and Engineering, Maastricht University, Maastricht, Netherlands; ^3^Instrumentation Engineering, Dean and Directors Office, Faculty of Psychology and Neuroscience, Maastricht University, Maastricht, Netherlands; ^4^Research Department, Brain Innovation B.V., Maastricht, Netherlands

**Keywords:** hemodynamic brain-computer interface, augmented reality, motor imagery, real-time analysis, temporal information encoding, user-centered approach

## Abstract

Augmented reality (AR) enhances the user’s environment by projecting virtual objects into the real world in real-time. Brain-computer interfaces (BCIs) are systems that enable users to control external devices with their brain signals. BCIs can exploit AR technology to interact with the physical and virtual world and to explore new ways of displaying feedback. This is important for users to perceive and regulate their brain activity or shape their communication intentions while operating in the physical world. In this study, twelve healthy participants were introduced to and asked to choose between two motor-imagery tasks: mental drawing and interacting with a virtual cube. Participants first performed a functional localizer run, which was used to select a single fNIRS channel for decoding their intentions in eight subsequent choice-encoding runs. In each run participants were asked to select one choice of a six-item list. A rotating AR cube was displayed on a computer screen as the main stimulus, where each face of the cube was presented for 6 s and represented one choice of the six-item list. For five consecutive trials, participants were instructed to perform the motor-imagery task when the face of the cube that represented their choice was facing them (therewith temporally encoding the selected choice). In the end of each run, participants were provided with the decoded choice based on a joint analysis of all five trials. If the decoded choice was incorrect, an active error-correction procedure was applied by the participant. The choice list provided in each run was based on the decoded choice of the previous run. The experimental design allowed participants to navigate twice through a virtual menu that consisted of four levels if all choices were correctly decoded. Here we demonstrate for the first time that by using AR feedback and flexible choice encoding in form of search trees, we can increase the degrees of freedom of a BCI system. We also show that participants can successfully navigate through a nested menu and achieve a mean accuracy of 74% using a single motor-imagery task and a single fNIRS channel.

## Introduction

A brain-computer interface (BCI) is a system that enables users to send commands to the external world through brain signals in the absence of motor output (Wolpaw et al., 2002). BCI research has mainly focused on developing applications for (1) changing brain activation and associated behavior voluntarily through neurofeedback (Subramanian et al., 2011; Scharnowski et al., 2012; Shereena et al., 2018) and for (2) replacing (lost) motor functions through communication BCIs (Birbaumer et al., 1999; Nijboer et al., 2008; Sellers et al., 2010) and (e.g., wheelchair/robotic body-part) control systems (Galan et al., 2008; Muller-Putz and Pfurtscheller, 2008; Iturrate et al., 2009; Rebsamen et al., 2011; Murphy et al., 2017). Independent of the application, information is fed back to users about the success or failure of the intended act (Leeb et al., 2007). In communication and control BCIs, feedback may allow the BCI user to adapt the communication content (of a next encoding trial) in a sense of “back-and-forth communication,” which enables users to communicate with or control a specific component of the external world.

The most common approach to provide feedback to users is through simplified unimodal (visual or auditory) representations of brain activation, such as bars or single tones (Sulzer et al., 2013). Alternative ways have emerged in the past years due to new technological developments in the areas of multimedia and entertainment, such as virtual reality (VR). VR is an immersive system that provides users with a sense of presence through potential interactions with a simulated virtual world rendered in real-time (Lécuyer et al., 2008). It has been suggested that VR environments can improve the BCI experience as it offers a richer and potentially more motivating feedback (Chin et al., 2010; Allison et al., 2012). Recent advances in VR research enabled the development of augmented reality (AR) systems. Unlike VR systems, AR enhances the environment the user is in by projecting virtual objects as overlays into the real world. This projection is called registration and it can be carried out using a camera that detects a number of fiducial markers placed in the real environment (Si-Mohammed et al., 2017). AR can be displayed using systems worn on the head (also known as head mounted displays, HMD) or visualized through a dedicated screen that the participant is not wearing (phone, computer screen, etc.). Depending on the augmentation type, AR systems can be divided into visual see-through (VST) and optical see-through (OST) systems. In VST-AR, real images are recorded in real-time by the camera of a device (tablet, phone, etc.) before being visualized through a screen, augmented with virtual information. In OST-AR, the virtual content is directly displayed in front of the user’s eyes onto a semi-transparent screen.

The number of studies exploring the use of BCIs in AR applications remains relatively small (Si-Mohammed et al., 2017). Up until now, the majority of the AR-BCI literature has focused on electroencephalography (EEG)-based evoked potentials applied to a wide range of fields, namely robotics (Lenhardt and Ritter, 2010), medicine (Blum et al., 2012), home automation (Takano et al., 2011; Park et al., 2019), navigation (Faller et al., 2010), and neurofeedback (Chin et al., 2010; Mercier-Ganady et al., 2014). Importantly, some of these studies have assessed the impact of AR feedback in mental workload and engagement compared to traditional forms of feedback. For example, Chin et al. (2010) compared 3D-AR displays vs. traditional 2D feedback (both displayed on a computer screen) and found that despite the higher mental load experienced by the participants during the 3D-AR feedback, participants reported the 3D-AR feedback being more engaging and motivating.

AR-BCIs based on hemodynamic signals have also been explored, but to a smaller extent (Si-Mohammed et al., 2017). One way of measuring hemodynamic signals is using functional near-infrared spectroscopy (fNIRS), a portable, silent, and affordable counterpart to functional magnetic resonance imaging (fMRI) (Scarapicchia et al., 2017). Both EEG and fNIRS make use of sensors [electrodes and optode pairs (sources and detectors), respectively] placed on the scalp to measure signals which correlate with neural activity (Allison et al., 2012). While EEG measures the postsynaptic potentials of ensembles of neurons, fNIRS is based on the optical measurement of the hemodynamic response of both oxy- and deoxyhemoglobin (HbO and HbR, respectively) to neural activity (Lloyd-Fox et al., 2010). Although EEG offers a higher temporal resolution than fNIRS, the latter represents an interesting option as it provides higher spatial resolution and is less vulnerable to motion artifacts (Lloyd-Fox et al., 2010).

To our knowledge, only three fNIRS-based AR-BCIs have been reported. Hu et al. (2019) used an fNIRS-based AR-BCI in a simulated real-time environment aimed at clinicians to measure and visualize in real-time the ongoing cortical activity to determine when and where the patients were suffering from pain. For that, they placed fNIRS optodes over the patients’ bilateral prefrontal cortex and primary somatosensory area and monitored brain activity while volunteers with hypersensitive teeth underwent a thermal stimulation session. The cortical activity was superimposed onto a participant’s head in the real world in real-time through an OST-HMD (*HoloLens)* device the clinician was wearing. Afergan et al. (2015) developed an fNIRS-based BCI using OST-HMD called *Phylter*. They developed a control system connected to *Google Glass* that helped preventing the user from getting flooded by notifications. By monitoring users’ mental workload in real-time with an fNIRS device, their system would only show notifications to the user if the mental workload was low enough. In the context of mental workload monitoring, McKendrick et al. (2016) assessed the cognitive differences between an AR wearable display (*Google Glass*) and a handheld display (smartphone) using a mobile fNIRS system covering the lateral PFC during an outdoor navigation task. They complimented it with two separate secondary tasks to assess differences in mental workload and situation awareness during navigation. They concluded that navigating with an AR wearable display produced the least workload during one of the working-memory task, and a trend for improved situational awareness in their measures of prefrontal hemodynamics. In this proof-of-concept study we tested whether healthy participants can use an AR fNIRS-based BCI paradigm motivated by the successful implementation in fNIRS-based BCIs, the increased engagement associated to the use of AR reported in previous studies (Chin et al., 2010) and its ability to preserve the real world while blending digital components to it.

Generally speaking, the hemodynamic response to a given task execution/stimulus shows a specific and reproducible temporal behavior (Menon and Kim, 1999). Previous fMRI-based BCI work exploited this property and demonstrated that up to four distinctive BCI commands could be encoded/decoded by varying the temporal aspects (onset, offset and/or duration) of a (set of) mental task(s) (Sorger et al., 2009, 2012; Bardin et al., 2011). Despite its simplicity, so far no fNIRS-based BCI has implemented this temporal information encoding approach. This is probably because the temporal encoding approach is serial in its nature, which can make the encoding process lengthy depending on the experimental design. In addition, it has been used in combination with univariate information decoding approaches, while the hemodynamic BCI community has mostly adopted multivariate classification techniques such as Linear Discriminant Analysis, Support Vector Machines or Artificial Neural Networks that have been used to exploit the spatial features of fNIRS signals evoked by performing different mental-imagery tasks (Naseer and Hong, 2015b; Hong et al., 2018). However, with appropriate experimental designs, the temporal encoding approach offers a way to increase the degrees of freedom of a BCI using a single mental task. With this in mind, the present study aimed at transferring the fMRI-based temporal encoding approach mentioned above to fNIRS. For that, we used a selection paradigm where participants had to sift through a multi-leveled menu using a motor-imagery task. This menu consisted of four levels, in such a way that the choice options provided in each level (always six) were based on the decoded choice of the previous level. Thus, here we expanded the traditional four-choice temporal information encoding approach to include six options for choice selection in each of the levels comprising the menu, where an AR object guided the temporal encoding approach. We then used a univariate procedure for decoding participants’ intention and used the same AR object to back-communicate the decoded answer of the participants’ intention. Additionally, to account for potential mistakes during the decoding process, we implemented an active error-correction procedure to be applied by the participants. Importantly, this specific combination of temporal encoding and univariate decoding approaches allows participants’ intentions to be decoded based on the information recorded from even a single fNIRS channel provided that this channel has enough signal quality. With this in mind, in the present study we used a single channel for decoding participants’ choices.

Although the application of BCIs has been limited primarily to a laboratory setting, some of the studies mentioned above have examined the possibility of using BCI in everyday-life settings in different contexts (Takano et al., 2011; Blum et al., 2012; Afergan et al., 2015; Hu et al., 2019; Park et al., 2019). However, ecologically valid approaches are challenging to develop as, among other reasons, they should be as efficient, accurate and reliable as possible, but also easy to use, intuitive, and simple to (dis)assemble. This is probably the reason why most BCI research has focused predominantly on improving the technology (Liberati et al., 2015). There is a relevant body of work addressing that BCI design and development should become more user-centered in order to achieve successful everyday-life applications (Kübler et al., 2014; Liberati et al., 2015; Nijboer, 2015). Effort has been made to incorporate this aspect into various applications (Weyand and Chau, 2015, 2017; Weyand et al., 2015; Nagels-Coune et al., 2017; Si-Mohammed et al., 2018). While still in a laboratory setting, in the present study we worked toward a user-centered communication system by letting participants choose their preferred motor-imagery task and by selecting participant-specific (single) most-informative fNIRS channel for decoding their choices. Using a single channel constitutes the simplest setup to (dis)assemble. In addition, it should make the setup comfortable and thus prevent participants from withdrawing from fNIRS recordings due to setup-related discomfort (Suzuki et al., 2010; Cui et al., 2011; Rezazadeh Sereshkeh et al., 2018).

It is important to note that fNIRS measurements are contaminated by systemic interference of especially (but not limited to) extracerebral regions, which is mainly caused by cardiac pulsations, respiration, and blood-pressure variations (Boas et al., 2004; Tachtsidis and Scholkmann, 2016). Several approaches have been reported in the literature to reduce these noises: conventional band-pass filtering (Hocke et al., 2018; Pinti et al., 2019); modeling physiological noises as a sum of sinusoidal functions with known frequencies where their amplitudes are estimated by using the extended Kalman filter and regressed out using a general linear model (Prince et al., 2003); global signal-covariance removal by either principal/independent component analysis (Zhang et al., 2005; Aarabi and Huppert, 2016) or global average procedures (Batula et al., 2017); adaptive filters that use recursive least-squares estimation methods (Nguyen et al., 2018) or short-distance channel (SDC) regression (Saager and Berger, 2005; Saager et al., 2011; Goodwin et al., 2014). In fNIRS measurements these SDCs are channels that have reduced inter-optode separations such that the interrogated volume is confined primarily to extracerebral regions (Goodwin et al., 2014). The main assumption underlying their usability is that the same systemic physiological noise present in the normal-distance channels (NDCs) dominates the signal acquired with SDCs (Gagnon et al., 2012). Intuitively, SDCs can then be used to minimize/reduce unwanted physiological noise from the normal-distance channels. So far, not many fNIRS-based BCIs have employed them (but see Shin et al., 2017). This is partially because fNIRS equipment that allows such measurements has only recently become widely available. Here, SDC correction was used for the selection of the most-informative fNIRS channel as well as during the decoding process.

In this preliminary study participants achieved mean accuracy level of 74% (with a chance-level of 37.5% for six answer options), which shows that the temporal features of the fNIRS signal can be exploited in a temporal encoding paradigm to increase the degrees of freedom of a BCI using a single mental task. These accuracies also indicate that the proposed fNIRS-based AR-BCI setup can be successfully controlled, on average, by participants. Importantly, this work conveys the fundamental steps toward developing the first fNIRS-based AR-BCI system to be used as a communication device for bedside applications in a clinical setting.

## Materials and Methods

### Participants

Twelve healthy volunteers [five males; mean age (*SD*) = 27.1 years (3.2 years)] with varying previous BCI/fNIRS/task experience participated in this study (see Table 1). Participants did not have a history of neurological disease and had a normal or corrected-to-normal vision. The experiment conformed to the *Declaration of Helsinki* and was approved by the ethics committee of the *Faculty of Psychology and Neuroscience*, *Maastricht University*. Informed consent was obtained from each participant before starting the measurements. Participants received financial compensation after the session.

**TABLE 1:** Participant characteristics.

			Previous experience			
			
	Age range	fNIRS Cap Size (cm)	BCI	fNIRS	Task	
					
					Mental drawing	Interacting with cube
P01	20–25	56	First time	<5 times	< 5times	First time
P02	20–25	56	<5 times	5–10 times	5–10 times	First time
P03	20–25	56	<5 times	< 5times	<5 times	First time
P04	25–30	56	>10 times	> 10 times	>10 times	First time
P05	35–40	58	First time	First time	First time	First time
P06	25–30	56	<5 times	<5times	5–10 times	First time
P07	25–30	58	<5 times	5–10 times	<5 times	First time
P08	25–30	58	First time	First time	First time	First time
P09	25–30	56	<5 times	< 5times	<5 times	First time
P10	25–30	56	5–10 times	5–10 times	5–10 times	First time
P11	25–30	56	>10 times	>10 times	>10 times	First time
P12	25–30	58	First time	<5 times	<5times	First time

### Experimental Design and Stimulus Display

#### General Structure

The experiment consisted of a training session and an immediately following experimental fNIRS session. The training session was self-paced and ranged between 15 and 35 min across participants: we only switched to the experimental fNIRS session when participants felt comfortable with the stimuli and the motor-task performance.

In an attempt to follow a user-centered approach, participants were introduced to two motor imagery tasks during the training session and asked to choose between them: (option 1) mental drawing [of small geometrical figures (a square, circle, etc.) or contour drawings (a star, flower, boat, etc.) and (option 2) imagine to interact with the virtually presented AR cube (by, e.g., to imagine to hit/squeeze it)]. Participants were asked to choose the mental task (mental drawing or imagining interacting with the cube), the specific strategy (drawing a square or imagining hitting the cube) they expected would work best and would interfere the least with the stimuli and to perform the motor-imagery task with their right hands. They were instructed to keep their eyes open throughout the experiment and to look at the computer screen while staying as still as possible during the runs.

**FIGURE 1:**
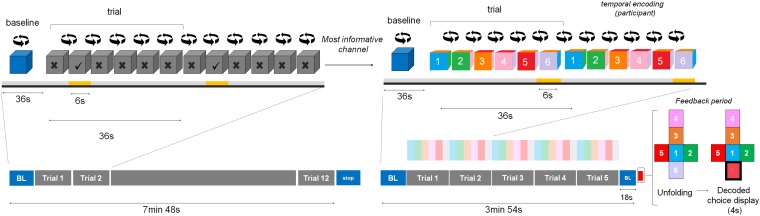
Experimental design. During the training session participants chose between two motor imagery tasks. Then, during the functional localizer run, participants performed the chosen task for twelve consecutive trials when the checkmark was facing them (indicated in yellow, below the face showing a checkmark) and had to rest for the remaining faces. There was an initial baseline (BL) period indicated by a blue rotating cube, in which participants rested. After the twelve trials the cube stopped rotating and became blue again, indicating signaling the end of the run (indicated with the word stop in the figure). The user-specific most-informative channel from this run was used to decode participants’ choices during the choice runs. Participants were asked to perform the mental task when the number corresponding to their choice was facing them (temporal information encoding), for five consecutive trials (in this example it corresponded to choice number 6, again underlined in yellow). After each run the feedback period started (indicated by the red square), where the cube unfolded and the decoded choice was highlighted in red (for visualization purposes, we added a black thick square in this schematic representation). After the choice runs, participants were asked to fill in several questionnaires.

The experimental fNIRS session lasted around 1.5 h. Participants first performed a functional localizer run, during which the participants were presented with a gray AR cube that contained specific symbols (5/6 = crosses, 1/6 = checkmark). For twelve consecutive times, they performed the selected motor imagery task when the checkmark was facing them (for 6 s) and had to rest for the remaining faces (for 30 s, see Figure 1). There was an initial baseline period of 36 s indicated by a blue rotating cube, in which participants rested. We chose a baseline period of 36 s to guarantee a stable baseline measure for real-time conversion of raw data into hemoglobin (Hb) concentration changes. After the twelve trials, the cube stopped rotating and became blue again, indicating the end of the run. This run was used to select a user-specific most-informative (“best”) fNIRS channel to decode participants’ choices in the eight subsequent choice-encoding runs (here on referred to as choice runs).

Each choice run aimed at selecting one option from a six-item list (menu). These runs differed from the functional localizer run in (1) the number of active motor imagery trials [five trials (choice runs) vs. twelve (functional localizer run)] and (2) the fact that the AR cube was color-coded and numbered (choice runs) vs. the AR cube was gray and contained geometrical shapes (functional localizer run). Importantly, the task duration remained at 6 s during the choice runs. During each choice run, participants selected one choice from a six-item list provided before the start of the run and performed the motor imagery task only when the number corresponding to their choice was facing them (temporal information encoding), for five consecutive times. There was an additional baseline period of 18 s after the last trial to ensure that the hemodynamic response goes back to baseline. After the run, the cube unfolded and the decoded choice (based on real-time analysis of the fNIRS data) was highlighted in red (see Figure 1).

#### AR Stimulus Display

**FIGURE 2:**
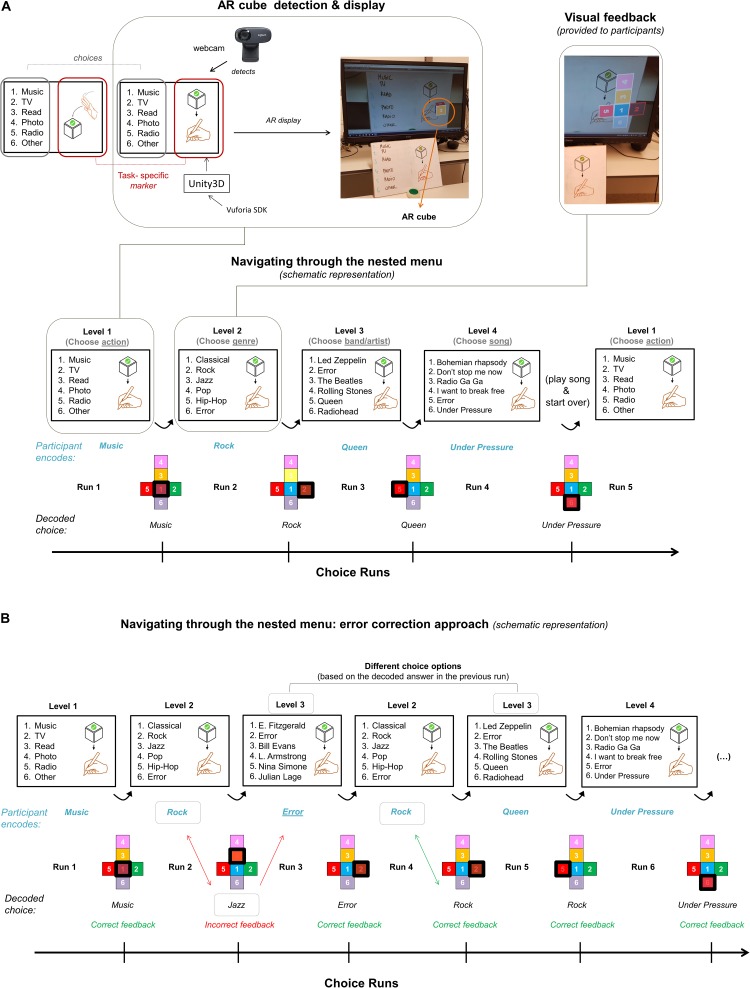
AR display and example of a full cycle of the nested menu. **(A)** A task-specific marker in the right-side of the A4 cardboard served as the spatial point of reference necessary for the visualization of the AR cube. This cube was used to navigate through a four-level nested menu with six options in each level. The choice options encoded by the participant are written in blue, while the decoded answers are written in black and highlighted in red with a black thick square in the schematic representation of the unfolded cube. The choice options provided in each level were based on the decoded choice of the previous run. **(B)** If the decoded choice was incorrect, they were asked to choose the “Error” option in the next run. If “Error” was decoded, they were provided with the same option list they saw before the error occurred. In this example, the participant chose to perform a mental drawing task, as indicated by the markers under “Navigating through the nested menu”. In the first level, we provided participants with keywords that responded to the question “What would you like to do?” Since the decoded choice [Listen to] Music (highlighted in red only in the actual run; highlighted in red and with a black thick square in the schematic view) was correct, the next run summarized music-genre options (Level 2). Here, the participant chose “Rock” [music] but the decoded choice was “Jazz”. Thus, the participant was provided with Jazz-band options in the next run (Level 3), where (s)he encoded the “Error” option. Since the “Error” option was correctly decoded (see displayed choice after Run 3), the participant was provided again with Level 2 choice options. The procedure went on until the participant reached the last level of the nested menu. At the end of the run, we played the decoded song (“Under pressure” in this example) to the participant and (s)he was directed back to the first level of the menu.

In this experiment, we used a variation of a VST-AR system, where a rotating AR cube displayed on a computer screen embodied the menu and each face of the cube represented one choice of the list (see Figure 2A for an example of a user’s view). In the presented AR system, a white A4 cardboard was used to represent the real-world stimulus that also served as a spatial point of reference necessary for the visualization of the AR cube. The A4 cardboard was placed on the desk, between the computer screen and the participants. The left half of the board was wrapped in transparent wrapping paper and served as a whiteboard, where choice options were handwritten (and modified after each run). The right half of the board contained a marker (a 2D-image, see hand-icon in top-left image of Figure 2A) that, when detected by the HD webcam (Logitech C270 HD, which was fixated on the participant’s forehead using an elastic band and recording the cardboard), triggered the visualization of the AR cube on a standard computer screen. The AR cube was placed relative to the marker as seen in the camera image (see top-left image in Figure 2A) with the help of Vuforia (v7.1.34), an AR software development kit (SDK) that was running in Unity3D. This SDK makes it possible to detect the marker and to place the virtual cube on it, creating the effect of augmented reality. The marker was motor imagery task-specific and reminded participants of the task to be performed (mental drawing or virtual interaction with the cube). After each run, an unfolded AR cube was displayed on the computer screen highlighting the decoded choice of the participant (see top-right image, Figure 2A).

#### Nested Menu and Error-Correction Approach

The menu presented during choice runs consisted of four levels that were interconnected in such a way that the choice options provided in each level were based on the decoded choice of the previous run. The provided answer options became more specific throughout the levels. An example transition of provided options from level one to level four would be: listen to music > choose a genre > choose a band/artist > choose a song. Displaying the selected choice of the fourth level (a song, a picture, a movie, etc., depending on the choice in the first level) indicated the end of the navigation round, and participants were directed back to the first level of the menu (see Figure 2A). This structure allowed participants to go through a four-level nested menu twice if all choices were correctly decoded.

Importantly, it could be that the decoded choice of any given level of the nested menu did not match the encoded option by the participant. To account for such decoding mistakes and in a first attempt to correct for it, participants were instructed to choose the “Error” option in the next run. This “Error” option was part of the choice list in levels > 1 and the position this option appeared on the menu list was balanced across the different levels. If “Error” was decoded, they were provided with the same option lists they saw before the decoding mistake was made (see first Level 2 trial in Figure 2B).

### fNIRS Data Acquisition

**FIGURE 3:**
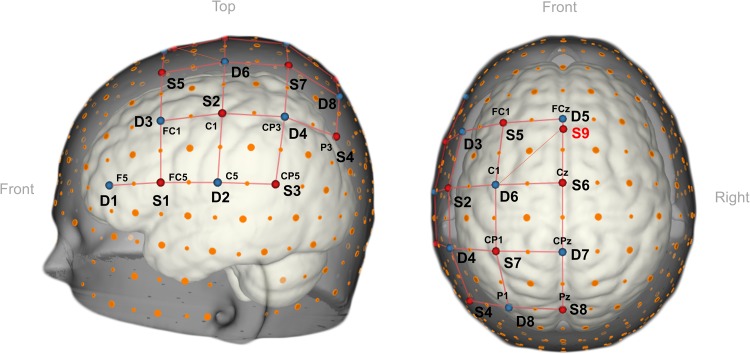
3D view of the fNIRS-optode arrangement. The setup consisted of nine sources (in red), eight detectors (in blue) placed over the left-hemipsheric motor and premotor regions. In total the setup contained one SDC (S9-D5) and 24 NDC. For the 3D representation we used NIRSite v1.0 software (NIRx Medizitechnik GmbH, Berlin, Germany; RRID: SCR_002491).

fNIRS data was recorded using a continuous-wave system (NIRScout-816, NIRx, Medizintechnik GmbH, Berlin, Germany). The optode setup consisted of nine sources and eight detectors which were placed on the left hemisphere that cover areas commonly associated with motor imagery, i.e., premotor cortex and part of the supplementary motor area, primary motor cortex, somatosensory motor cortex and part of the parietal cortex following the extended 10/10 EEG system (see Figure 3; Sorger et al., 2012; Abdalmalak et al., 2016; Batula et al., 2017; Klein and Kranczioch, 2019; Erdogan et al., 2019). An in-house SDC was created by placing source S9 as close as the optodes would allow (∼13 mm away) to detector D5 on the same sagittal plane that connects D5 and source S6 (see Figure 3). The signal measured by the SDC should be influenced by the mid-sagittal sinus and other large vascular structures commonly found in this region (Duvernoy et al., 1981), which have been shown to be affected by low frequency oscillations and cardiac signals (Tong and Frederick, 2012). We used this information as a proxy to account for physiological noise in the region covered by the optode setup.

In total, the setup contained 24 NDCs and one SDC. The mean inter-optode distance of the standard channels spanned from 26.1 to 36.5 mm. Sources emitted light at wavelengths 760 and 850 nm, and the light intensity acquired at the detector side was sampled at 6.94 Hz. Besides the standard cap fixation (using the chin band), the fNIRS cap (EasyCap 128Ch ActiCap, EasyCap GmbH, Herrsching, Germany) was fixated onto the participants’ head with three medical tape stripes (connecting the cap and the participant’s forehead) to assure the cap would not shift during the measurements. In addition, a black, plastic overcap was placed on top of the fNIRS cap to additionally prevent the light in the room from reaching the optodes.

### Apparatus

**FIGURE 4:**
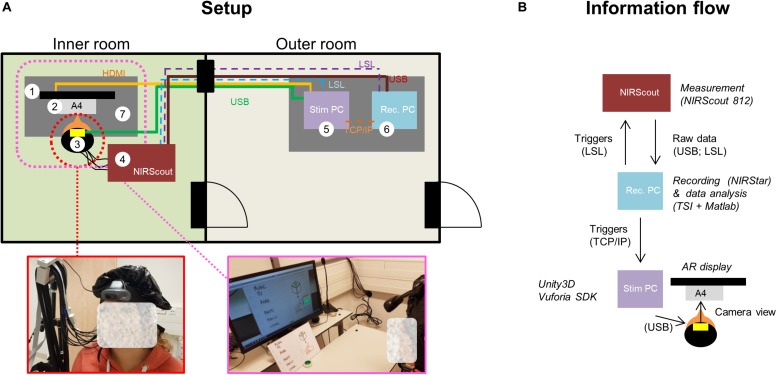
Summary of the technical setup and connections between its different components. **(A)** Setup. The inner room, where participants were measured while seated (see (3) and enlarged picture), contained the fNIRS system (4), a computer screen (1), an HD webcam (3), the A4 cardboard (2) and a desk (7). The outer room, where the experimenter was located, hosted the two laptops, i.e., the data-recording and -analysis laptop (6) and the stimulus laptop (5). Physical connections (wires) are depicted with continuous lines, while non-physical connections [Lab Stream Layer (LSL), TCP/IP] connections are depicted with dashed lines. **(B)** Information flow. NIRStar 15.2 was connected to the NIRScout system via USB and to Turbo-Satori (TSI) viaLSL. TSI and Matlab were connected via the TSI-Matlab interface. Matlab was used to send triggers back to the fNIRS system via LSL and to control the stimulus display in Unity3D software (via TCP/IP).

The session took place in a lab that consisted of two rooms, i.e., an inner and an outer room, where the hardware and materials comprising the setup were distributed (see Figure 4). We used NIRStar 15.2 (NIRx, Medizintechnik GmbH, Berlin, Germany) for recording the data and Turbo-Satori (TSI) 1.4.2 (BrainInnovation B.V., Maastricht, the Netherlands; Lührs and Goebel, 2017) and Matlab 2017a (The MathWorks Inc., Natick, Massachusetts, United States) for real-time preprocessing and decoding the participants’ choices, respectively (see Data Analysis section). The three programs ran on the data-recording and -analysis laptop (depicted with number 6 in Figure 4). NIRStar 15.2 was connected to the NIRScout system via USB and to TSI via Lab Streaming Layer (LSL). TSI and Matlab were connected via the TSI-Matlab interface, a self-designed network interface enabling real-time access to raw and preprocessed fNIRS data as well as protocol and statistical information (Lührs and Goebel, 2017; BrainInnovationSupport, 2019). In addition, Matlab was used to log the different experimental conditions by sending triggers to the fNIRS system via LSL and to control the stimulus display in that was running in Unity 3D software (v2018.3.2.f1, Unity Technologies, San Francisco, California, United States), which was running in the stimulus laptop (number 5 in Figure 4). During choice-encoding runs Matlab sent to Unity3D the following commands via TCP/IP: (“a”) start of the run, which initiated the rotation of the inactive (blue) AR cube; (“b”), start of the encoding period, which turned the inactive cube into an active one by changing the blue-colored faces into color-coded faces; (“c”) last rest period, which turned the face of the AR cube back to blue, indicating the last rest period of the run; (“1–6”) decoded choice, which unfolded the cube and highlighted in red the decoded choice. All commands except for those pertaining to the decoded choice were used for the functional localizer run. The computer screen in the inner room was connected to the stimulus laptop through an HDMI cable. The HD webcam in the inner room (number 3 in Figure 4) was connected to the stimulus laptop via USB.

### Subjective Ratings and Previous Experience Report

After the completion of the experiment, participants first rated how comfortable the setup (optodes and webcam) felt throughout the session according to a Likert-scale ranging from 0 (extremely uncomfortable) to 10 (extremely comfortable). We predicted that comfortability ratings would decrease over time due to the presence of local pressure on the head surface caused by optodes (Nagels-Coune et al., 2017) and the webcam. Then participants rated the general easiness, pleasantness and vividness of the two motor imagery tasks they were trained on using another Likert-scale ranging from 0 (extremely difficult/unpleasant/not vivid at all) to 10 (extremely easy/pleasant/very vivid). In addition, participants were asked to report on their previous motor imagery task, fNIRS and BCI experience (first time, less than five, five to ten times or more than ten experiments).

### Data Analysis

#### Real-Time Analysis

##### Data preprocessing

Raw fNIRS data were first converted into optical-density data and then into changes in Hb concentration through the modified Beer-Lambert law in real-time, using differential path-length factors of λ_760_ = 6.40 and λ_850_ = 5.85 (Essenpreis et al., 1993) and a baseline calculation period of 15 s (10–25 s after run onset). Data were filtered using a first-order moving-average high-pass filter with a cutoff of 0.01 Hz and a second-order moving-average low-pass filter with a cutoff of 0.25 Hz. No motion correction was applied.

##### Channel selection

The channel and Hb-type selection per participant was based on the result of the general linear model (GLM) analysis. Specifically, the selection was based on the chromophore and channel that led to the highest *t*-statistic of the task vs. rest contrast in the functional localizer run. The design matrix included one task predictor convolved with a standard hemodynamic response function (HRF). The default HRF from SPM12 was used (two Gamma HRF, the onset of response and undershoot 6 and 16 s, respectively, dispersion 1 s, response to undershot ratio 6) and the same amplitudes were used for the HbO and HbR task predictors. In addition, a constant term and the SDC time course were used as confound predictors should the latter satisfy the coefficient of variation criterion (CV < 7.5%, which was the case for all participants). The pre-whitening approach implemented in TSI was used to remove serial correlations (Lührs and Goebel, 2017).

##### Temporal decoding

**FIGURE 5:**
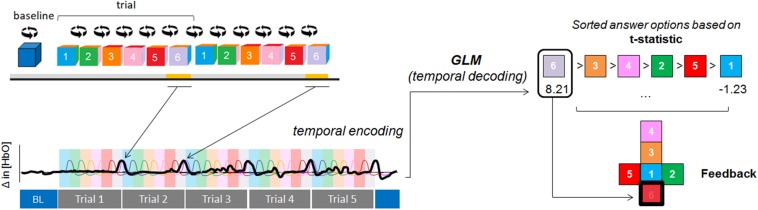
Temporal-decoding approach. A GLM was fitted to the HbX data (from five repeated trials) to decode the participants’ intentions. In this example, the participant encoded option #6 (represented by the black, thick line) and HbO signal was used for decoding. Each colored area represents the encoding time (the period where participants were instructed to perform the mental task) for each of the cube faces. Each colored HRF represents the expected fNIRS response for each of the options. After the run the cube unfolded and feedback was provided by highlighting in red the decoded intention (which was the condition that led to the highest *t*-statistic [option 6, *t*-value = 8.21]). For visualization purposes, we added a black thick square in this schematic representation).

During choice runs the time course of the selected channel was read in real-time in Matlab using the TSI-Matlab interface. Participants’ choices were decoded by fitting a GLM in Matlab using *glmfit* to all five trials in each choice run (see Figure 5). The design matrix differed from the functional localizer run in that it included six task predictors (one for each choice option, i.e., choice period) instead of one convolved with the HRF. Importantly, the SDC time course was used as a confound predictor during choice runs only if it was used as a confound predictor during the channel selection process. No pre-whitening was applied. The condition that led to the highest *t*-estimate of the task vs. rest contrast was considered the selected choice (see Figure 5). It should be noted that this analysis was re-computed offline using a simulated real-time approach for participants P01–P07 due to a technical mistake during these sessions.

#### Offline Analysis

##### Channel-selection assessment

We evaluated the effect (on choice-decoding accuracies) of using a predefined Hb type for the channel selection vs. selecting the most informative HbX channel (where HbX ε {HbO, HbR}). Importantly, and despite following a single-channel decoding approach, we kept all channels in place to carry out this assessment.

Besides, we evaluated the effect (on choice-decoding accuracies) of using the SDC as confound predictor in the channel-selection process. Differences across Hb-type and usage of SDC were tested for significance using a two-way ANOVA with factors SDC (with SDC, without SDC) × Hb-type (HbX, HbO, HbR), followed by paired *t*-tests.

##### Effect of the number of trials in the decoding process

We used the same univariate choice-decoding approach as described in section Temporal decoding to evaluate the effect of the number of trials in a given run on decoding accuracies (based on the most informative HbX channel). For that, we computed the accuracies of all consecutive trial combinations for every trial number (1:*n* trials, where *n* = {1,2,3,4,5}). For example, to compute the decoding accuracy of three trials, trial combinations 1-2-3, 2-3-4, and 3-4-5 were used. We then quantified the effect of the number of repetitions in the decoding accuracy at the group level using Spearman’s rho correlation coefficient. The effect of number of trials was additionally evaluated using information transfer rate (ITR), defined as in Allison et al. (2012):

(1) $$I\,T\,R={\left(l\,o\,g_{2}\,N+P^{*}\,l\,o\,g_{2}\,P+{\left(1-P\right)}^{*}\,l\,o\,g_{2}\,\left(\frac{1-P}{N-1}\right)\right)}^{*}\,\frac{60}{\tau }$$

where N is the number of classes, P is the classification accuracy and τ is the duration of task and rest period, in seconds.

##### Decoding accuracy of error-correction trials

We incorporated an error-correction mechanism in our decoding process by including an “Error” option in levels > 1 of the menu. We assessed the accuracy of the error-correction approach with a confusion matrix. For that, we pooled all encoded answers across participants and divided them into “Error” and “Non-Error” instances, depending on whether the participant intended to encode “Error” or not, respectively. The encoded choices were then compared to the decoded ones. Four measures were extracted from the confusion matrix, namely accuracy, recall, precision and specificity, which were calculated as follows:

• Accuracy = (TP + TN)/(TP + TN + FP + FN)
• Recall = TP/(TP + FN)
• Precision = TP/(TP + FP)
• Specificity = TN/(TN + FP)

where TP = True positive or correctly detected “Error” trials; TN = True negative or correctly detected “Non-Error” trials; FP = False Positive or incorrectly detected “Error” trials; FN = False negative or incorrectly undetected “Error” trial.

##### Chance-level definition

A quantile function of a multinomial distribution was used to define the upper bound of the chance-level (37.5% for N = eight runs, c = six classes and a *p* < 0.05).

##### Subjective ratings

Mean and SE of normalized subjective comfortability ratings was computed by calculating the mean (of eight runs) for each subject and subtracting the subject’s mean to each item. The effect of the duration of the experiment (number of runs) on the comfortability score was quantified using Pearson’s correlation. In addition, the relation between previous BCI/fNIRS/task experience on task accuracies reached by each participant was assessed using Spearman’s correlation coefficient. Finally, to evaluate the perceptual differences the mental tasks elicit on each participant, normalized absolute mean differences between the preferred and non-preferred mental task ratings were assessed. First, each item was normalized following the same approach as for the comfortability ratings. Next, the three scores (easiness, pleasantness and vividness) were averaged for each mental task and participant. Then, absolute differences between mental tasks were computed and a right-tailed *t-*test was used in Matlab.

## Results

### Choice-Decoding Results Obtained in (Simulated) Real-Time

**FIGURE 6:**
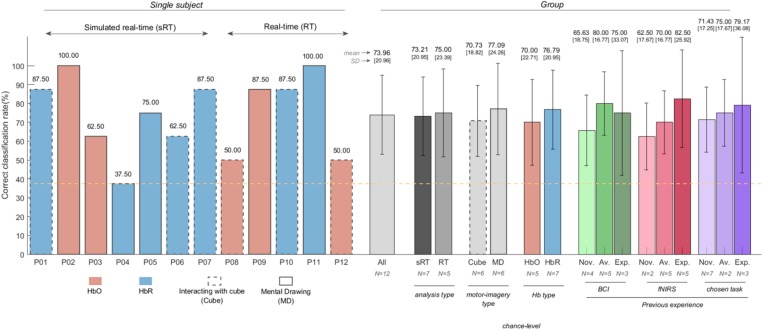
Choice-decoding accuracies obtained in (simulated) real-time (individual and group results) using HbX channel selection and SDC correction. All participants but P04 reached accuracies higher than chance-level (orange, dashed line). The face colors and line pattern of the bar plots of each subject (left-half of the figure) represent the selected Hb-type and strategy participants chose to perform, respectively. Mean decoding accuracies and standard deviation of all participants and with different grouping factors can be found on the right-half of the figure. Groupings were based on the analysis type (simulated real-time vs. real-time), the motor imagery participants chose [interacting with the cube (cube) vs. mental drawing (MD)], the selected chromophore (HbO vs. HbR) and previous BCI experience (novices, average and experts). Participants with no previous BCI/fNIRS/task experience were considered *novices*; *expert* participants were those who had participated in more than five BCI experiments; the remaining participants were considered *average* (see Table 2). The integer after “*N* = ” indicates the number of participants employed in each computation.

Figure 6 shows the individual and group accuracies achieved in the experiment. In addition, it shows that half of the participants chose to perform the mental-drawing task and that HbR was selected for seven out of twelve participants. All participants but P04 exceeded the upper bound of the chance-level (37.50%, orange dashed line). It should be noted that accuracies from participants P01-P07 were computed offline using a simulated real-time approach due to a technical mistake during these sessions, while accuracies from participants P08-P12 were calculated online based on real-time results. On average, participants reached an accuracy of 73.96% (*SD* = 20.96), as depicted by the left-most gray bar of the group plots. Mean decoding accuracies with different grouping factors were also computed and descriptively did not differ substantially within each group (see Figure 6).

### Evaluation of Error-Correction Procedure

**FIGURE 7:**
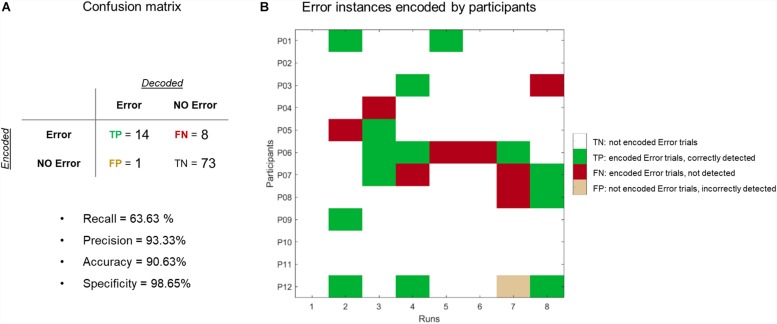
Evaluation of error-correction procedure. **(A)** Confusion matrix. We reached an accuracy of 90.62% (72/96 trials were correctly labeled as “Error” or “NoError”) and a recall level of 65.22% (out of 22 error trials, 8 trials were missed). **(B)** Summary matrix of when participants encoded the “Error” option (marked in dark gray). Green (red) cells represent trials where the “Error” option was correctly (incorrectly) detected. Beige cells indicate a false positive trial.

In total, participants had to encode the “Error” option 22 times (see Figures 7A,B). Out of the 22 instances, the error option was correctly detected 14 times, missed eight times, and incorrectly labeled once, as indicated in the confusion matrix (Figure 7A). Overall, the accuracy of the error-correction trials was 90.6% (upper bound of the chance level was 58.88%, assessed by the quantile function of a multinomial distribution with *n* = 96 trials, *c* = 2 classes and alpha = 0.05).

### Assessment of the Effect of Number of Trial Repetitions

**FIGURE 8:**
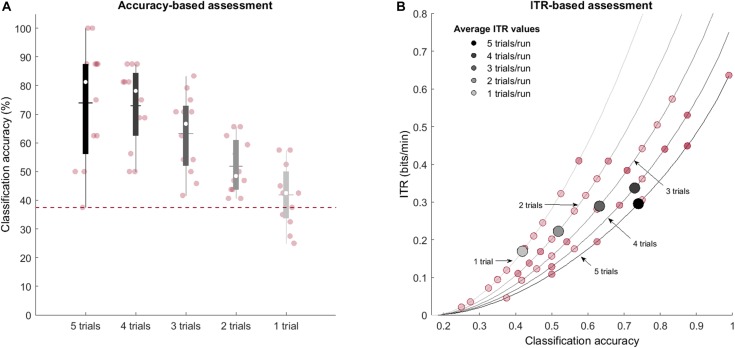
Effect of the number of trial repetitions on obtained decoding accuracy (individual and group results). **(A)** The box-plot shading depicts the number of repetitions used for decoding: from five trials (black) to a single trial (light gray). Median values are represented by the white circles, while the mean values are indicated with the horizontal lines. The y-axis represents the accuracy (%) achieved by the participant. The red, dashed line shows the chance-level defined by the cumulative multinomial distribution. The number of trials used to decode each run influences the decoding process, but mean- and several single-subject accuracies remain above chance level even with a single trial. **(B)** Average (gray-scale markers) and single-subject (red markers) ITR values (bits/min) for different number of trials as a function of achieved classification accuracies. Lines represent the theoretical values the ITR can take as a function of the number of classes, trial duration and accuracy.

To assess how the number of trial repetitions affects the decoding process, we sequentially reduced the number of trial repetitions we used for decoding. Table 2 summarizes the individual and group decoding accuracies for a decreasing number of repetitions and Figure 8A shows that the number of repetitions used to decode each run influences the decoding process. Specifically, we observed a significant negative correlation between the accuracies and the number of repetitions, as assessed by Spearman’s rho correlation coefficient (ρ = −0.639, *p* < 0.0001). Importantly, mean- and several single-subject accuracies (7 out of 12 participants) remain above chance level even when using a single trial. As for the ITR computation, Figure 8B indicates that slightly higher ITR values can be reached, on average, when using four trials (0.34 bits/min) instead of five (0.29 bits/min).

**TABLE 2:** Individual and group decoding accuracies over decreasing number of repetitions (for HbX with SDC regression analysis).

	Accuracies (%)				
	
	5 trials	4 trials	3 trials	2 trials	1 trials
P01	87.50	81.25	70.83	65.63	47.50
P02	100.00	81.25	70.83	59.38	57.50
P03	62.50	75.00	54.17	56.25	45.00
P04	37.50	50.00	54.17	43.75	37.50
P05	75.00	50.00	50.00	50.00	42.50
P06	62.50	68.75	41.67	46.88	27.50
P07	87.50	87.50	79.17	65.63	57.50
P08	50.00	56.25	45.83	40.63	32.50
P09	87.50	81.25	75.00	43.75	35.00
P10	87.50	87.50	83.33	62.50	52.50
P11	100.00	87.50	62.50	46.88	42.50
P12	50.00	68.75	70.83	40.63	25.00
Group (SD)	73.96 (20.96)	72.92 (14.19)	63.19 (13.74)	51.82 (9.56)	41.88 (11.83)

### Assessment of Channel Selection

Although our channel selection approach was based on selecting the most informative HbX channel for each participant, it is not uncommon to have a predefined Hb-type before the data acquisition (Naseer and Hong, 2015b). In this context, we looked at whether the selected channel would change had we decided to focus on only one chromophore. In addition, since we used the SDC time course as a confound predictor, we assessed whether applying SDC correction (or not) influences the channel selection. Table 3 shows that for some participants, the channel selection approach does not affect the selected channel (see P01, P02, P07 and P11 across all columns), while for other participants it does. Descriptively speaking, SDC correction slightly reduced the mean accuracy for the most-informative HbX-channel approach. The reason behind this observation is that the increased accuracy for some participants (P03, P06, P09, P11, and P12) was smaller than the decrease in accuracies for other participants (P04, P05, P08, and P10). The mean decoding accuracy increased for the most-informative HbO and HbR channel approaches (although to a considerably lesser extent for the latter).

**TABLE 3:** Most informative channel for different channel selection approaches and (individual and mean) accuracies reached with each approach.

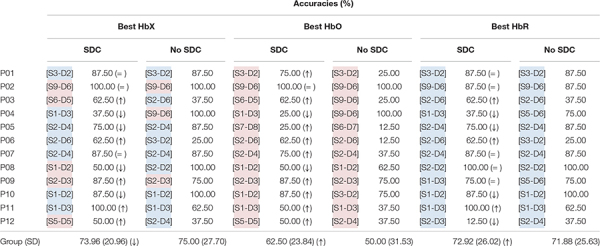

*Note 1: Red (blue) cells indicate that the selected chromophore was HbO (HbR) *Note 2: The different symbols summarize the effect in decoding accuracy (↑ [increased], ↓ [decreased], = [maintained]) when SDC was used as a confound predictor vs. when it was not.**

A repeated measures 2-way ANOVA with factors SDC (with SDC, without SDC) × Hb-type (HbX, HbO, HbR) showed that the mean accuracies were different across Hb-types [main effect of Hb-types; *F*_(__2_,_66__)_ = 3.494, *p* = 0.036; no significant interaction], but not across SDC. Subsequent paired *t*-tests showed that HbX and HbR performed better than HbO [*t*_(__23__)_ = 3.83; *p*(FDR [*q* = 0.05]) = 0.001, and *t*_(__23__)_ = 2.736; *p*(FDR [*q* = 0.05]) = 0.008].

### Previous Experience and Subjective Reports

Due to the (novel) AR component, the participants were enthusiastic about the research study. Independent of the achieved accuracies participants rated the setup positively and considered the experiment as “fun,” “engaging,” and “motivating.” The setup became uncomfortable over the runs as indicated by a significant negative correlation (*r* = -0.991, *p* < 0.0001). Participants reported the main source of discomfort to be the pressure caused by the webcam on their foreheads and to a lesser extent the optodes on the head surface. We observed that the preferred motor imagery task was rated significantly higher than the non-preferred task [*t*_(__11__)_ = 5.240, *p* < 0.001]. In addition, we observed that previous BCI/fNIRS/task experience correlated positively with individual accuracies, but none of them reached significance (ρ_task_ = 0.429, ρ_BCI_ = 0.360, ρ_fNIRS_ = 0.566, *p* > 0.05).

## Discussion

The present proof-of-concept study combined AR technology and an fNIRS-based BCI to apply it in a communication context, where twelve healthy participants were asked to navigate in real-time through a nested six-choice menu while following a temporal information encoding approach. The decoded choice was defined for each participant based on the time course of the most-informative channel in the setup. In case the decoded choice was incorrect, an active error correction procedure was used. We achieved mean accuracy levels of 73.96% (with a chance-level of 37.5% for six answer options) and error detection accuracies of 90.6%. The following sections discuss the general implications of this study, together with its limitations and prospects for the future.

### The Temporal Information Encoding Approach – A Powerful Paradigm for fNIRS-Based BCIs

In this experiment, we applied for the first time a temporal information encoding approach and a GLM-based decoding scheme previously reported in fMRI-based BCIs (Sorger et al., 2009, 2012; Bardin et al., 2011) to an fNIRS-based BCI system to distinguish between six options using a single channel and mental task. An advantage of using this procedure is that a single channel may be sufficient for decoding participants’ intentions without hampering our decoding ability. Intuitively, using a single channel should also make the setup more comfortable. It should be mentioned that although we assessed the feasibility of the single-channel approach and recorded participants’ comfortability scores over time, we kept all channels in place for *post hoc* analyses. Another advantage is that, theoretically, this approach could allow including a considerably high number of conditions. In the present work we have further advanced previous applications by going from four (Sorger et al., 2009; Bardin et al., 2011) to now six temporally different but still differentiable encoding phases. Importantly, future work should explore the upper limit of the included number of conditions that would yield a sufficiently high decoding accuracy. In any case, increasing the number of conditions would inevitably rise the duration of the run, but this could be solved by reducing the task duration to a certain extent. Until now the biggest body of hemodynamic BCI applications has used a task duration of 10 s (Naseer and Hong, 2013; Herff et al., 2013; Hong and Santosa, 2016; Nagels-Coune et al., 2017; Shin et al., 2017; Snipes et al., 2017) or longer (Bardin et al., 2011; Bauernfeind et al., 2011; Batula et al., 2017), and very few studies have used task durations under 10 s: for example, Sorger et al. (2009) and Shin and Jeong (2014) used variable task durations of 5/10/15 s and 6/8/10/15 s, respectively. To maintain the single-trial duration as low as possible without hindering the ability to distinguish between conditions, we opted to use 6 s task duration per condition for our experiment. However, the considerable inter-subject variability in accuracies suggests that user-tailored task durations should be considered in future studies.

### Using a Single fNIRS Channel – A Promising Approach in the Context of Temporal Information Encoding

#### Selected Feature

Feature selection varies across studies, but in general, previous work has focused on either using only HbO signal (Stangl et al., 2013; Erdoğan et al., 2014; Hong et al., 2015; Koo et al., 2015; Hong and Santosa, 2016; Lapborisuth et al., 2017; Noori et al., 2017; Liu et al., 2018) or the combination of different chromophores (computing the mean or the difference of HbO and HbR, Naseer and Hong, 2015b). A few fNIRS-BCI applications have used/explored HbR on its own (Cui et al., 2010; Naseer and Hong, 2015a; Hwang et al., 2016). The main reason is that HbO is considered to exhibit larger and more pronounced concentration changes than HbR in response to mental tasks (Stangl et al., 2013; Sato et al., 2016). Besides, it has been reported that HbO signals are more discriminative and perform more robustly than HbR signals (Mihara et al., 2012; Naseer and Hong, 2015a). However, Cui et al. (2010) and Hwang et al. (2016) found that HbO and HbR performed similarly in terms of accuracy. In the present work the channel selection approach led to selecting HbR for 7/12 participants. In addition, our *post hoc* analysis revealed that at the group level channel selection using either HbX approach or HbR performed better than only HbO channel selection. Despite having lower SNR, these results point at the usefulness of the HbR signal for the classification of motor imagery (at least) in a GLM-based decoding approach.

#### SDC Correction

SDCs are used to minimize/reduce unwanted physiological noise contained in NDCs (Goodwin et al., 2014). In the current work, a custom-built SDC was used as a GLM confound predictor during both, the selection of the most informative channel and the decoding process. Offline, we evaluated the effect of using SDC for channel selection and choice decoding. As derivable from Table 3, when using the HbX approach, SDC correction did not affect the channel selection in seven out of twelve participants (P01, P02, P05, P07, P09, P10, and P11). The selected channels for the remaining participants differed either in location only (P06) or in location and Hb-type (P03, P04, P08, and P12). This suggests that the former group of participants had a relatively stable signal compared to the latter ones. Interestingly, the mean accuracies were higher for the former group, too [89.29% (*SD* = 8.63) vs. 58.33% (17.08)]. Although the accuracy did not significantly change on average when SDC correction was used vs. when it was not, a clear divergence between both approaches was observed in some participants. For example, the accuracy reached by P04 and P08 was considerably reduced after SDC correction (100–37.5% and 100–50%, respectively), while it improved for P06 and P11 (25–62.5% and 62.5–100%, respectively). It is not straightforward to attribute this opposing and seemingly irregular effect across participants to an isolated cause. Instead, it may be the result of an interaction between the spatial relation of the SDC and the selected channel, which suggests that the location of the SDC matters even in a relatively small setup. In addition, the selected chromophore (whether it is HbO or HbR) may influence the effect of SDC correction. Indeed, unlike for the HbX (and the HbR) approach, we observed a clear improvement before/after SDC correction when selecting channels based on HbO (see Table 3, “Best HbO”). Specifically, the mean decoded accuracy increased from 50 to 62.5% after SDC correction. This is expected, as HbO signal is more affected by global systemic artifacts in both extracerebral and intracerebral compartments than HbR (Kirilina et al., 2012).

#### *T*-Statistic for Channel Selection and Decoding

Different approaches for channel selection have been reported in the literature. Hu et al. (2013) compared the difference between the maximum value during the task and rest periods, and considered the channel to be active if the difference was positive. Hong and Naseer (2016) and Khan and Hong (2017) suggested selecting channels where the initial dip could be reliably detected. For that, a vector-based phase analysis with a threshold circle as a decision criterion was employed. Previous fNIRS studies have also followed a *t*-value (Hong and Santosa, 2016; Nagels-Coune et al., 2017) or beta-value criterion (Klein and Kranczioch, 2019) between the measured and expected hemodynamic response by the given stimulation for channel selection.

In the present study we selected the most informative channel and Hb-type combination based on the highest *t*-statistic of the task vs. rest contrast of the functional localizer data. We ensured correct *t*-value estimation during channel selection by removing serial correlations generally present in the fNIRS data (Huppert, 2016). The decoded answer option was based on the choice that led to the highest *t*-statistic of the choice*_*i*_* vs. rest contrasts, where i = {1,2,3,4,5,6}. No pre-whitening was used during decoding since the ranking of the *t-estimate* should not change across choices. The reason for this is that, as a single channel was used for decoding, each *t-estimate* was affected by the same amount of serial correlations (Lührs et al., 2019).

### Necessity of Trial Repetition

Sorger et al. (2009) and Bardin et al. (2011) used an fMRI-based temporal-encoding and decoding approach to carry out five and two communication runs (respectively) with four answer options; while Sorger et al. (2012) used it in a letter speller context with 27 letter options to encode words between 7 and 13 characters. They reached single-trial mean accuracies of 94.9% (Sorger et al., 2009), 100% (Bardin et al., 2011), and 82% (Sorger et al., 2012) in healthy participants. As for fNIRS-based BCIs, previous work has addressed classification problems using multivariate approaches that maximally distinguished between five mental tasks with an average single-trial accuracy of 37.2% (Weyand and Chau, 2015), or four commands involving motor-execution (Shin and Jeong, 2014) and motor imagery tasks (Batula et al., 2014; Naseer and Hong, 2015a; Weyand and Chau, 2015) that reached mean single-trial accuracies of 82.46, 45.6, 73.3, and 46.7%, respectively. In the present work, participants encoded the same choice five consecutive times in each of the eight choice runs, and we achieved mean (multi-trial/repetition) accuracy levels of 74%.

**FIGURE 9:**
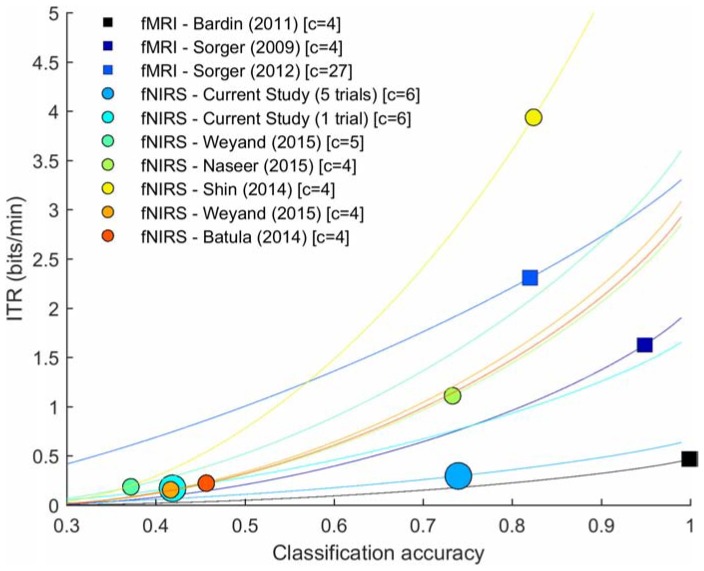
Average ITR values from relevant hemodynamic-BCI literature. Square markers represent fMRI-based BCIs, while circular markers represent fNIRS-based BCIs. Lines depict the theoretical values the ITR (bits/min) can take as a function of the number of classes (c), trial duration and accuracy.

To assess whether five consecutive trials were actually necessary to successfully decode their choice, the effect of reducing the repetitions on the decoding accuracy was evaluated *post hoc*. We observed a significant negative correlation between the accuracies and decreasing the number of repetitions (ρ = −0.639, *p* < 0.0001). Interestingly, encoding the same choice only once maintained the mean group accuracies above chance level although with considerably lower values than with five trials (73.96% vs. 41.88%). In line with the observed accuracies, the mean ITR value was considerably reduced when a single trial is used (ITR_l_ = 0.17 bits/min) compared to when five trials were used (ITR_5_ = 0.30 bits/min). In addition, we observed that reducing the number of repetitions to four slightly improves the mean ITR, with 0.34 bits/min. To put these values in a broader context, the average ITR of the studies mentioned above were calculated and can be compared to the present study in Figure 9. This figure shows that the ITR_1_ is closely related to the ITR values from Batula et al. (2014) and Weyand and Chau (2015) and that with the approach employed in this study (ITR_5_) considerably higher accuracies are reached, while maintaining the ITR value. This figure also depicts that ITR_5_ is considerably lower than in Shin and Jeong (2014) and Naseer and Hong (2015a).

Lower decoding accuracies compared to fMRI studies are expected since fMRI has a higher spatial resolution (Valente et al., 2019), fMRI signals have stronger signal-to-noise ratio (Cui et al., 2011) and because unlike fMRI, the brain signal measured with fNIRS also contains (unwanted) superficial scalp information (Erdoğan et al., 2014). This is because light traveling from a source to a detector to reach the brain must pass through scalp and skull tissues twice (Brigadoi and Cooper, 2015). Lower decoding accuracies compared to fNIRS-BCI studies employing multivariate approaches may require further explanation. Multivariate approaches are pattern-classification algorithms used to decode the information that is represented in a given pattern of activity (Norman et al., 2006). They integrate information of multiple voxels/electrodes/channels by optimizing their weights and theoretically should provide higher sensitivity to disentangle overlapping distributed activation patterns than univariate approaches (Valente et al., 2019). The fundamental steps comprising multivariate approaches are, generally speaking, feature extraction, feature selection, model learning and validation (Norman et al., 2006). The available number of trials/examples for model learning and feature extraction influences the performance of multivariate approaches, as estimating a model based on few examples may not be sufficiently reliable or may not capture the differences between classes in a relatively high-dimensional space (Valente et al., 2019). Thus, it is expected that a model trained on a sufficient number of examples should be able to accurately classify examples never seen by the model. Naseer and Hong (2015a) and Shin and Jeong (2014) employed multivariate approaches and both used > 100 trials to train their models, collected over four separate sessions and a single session, respectively. In addition, their classification problem aimed at distinguishing between different task patterns, which we suspect may elicit more discernible patterns than classification problems aimed at detecting the presence or absence of a task-related information (i.e., task vs. rest scenario). It should be noted that Batula et al. (2014) and Weyand and Chau (2015) also applied multivariate approaches that aimed at distinguishing between different motor imagery tasks, but employed less total number of trials to address the classification problem, which can partially explain the lower accuracies reported in these studies. The temporal approach employed in this study did not require any model learning, but relied on a time course extracted from a single channel with certain degree of trial-to-trial variability that was not constant across participants. Indeed, in some participants (see P03, P04, P06, P08, or P12), we did not observe a linear decrease in the decoding performance with reducing the number of repetitions as in the group results, which suggests that for some participants the inter-trial variability is higher than for others. Altogether, we believe these are the main reasons that could explain the divergence in the mean single-trial accuracies observed in the present study and in the literature.

In the future, a multivariate temporal approach could be tested that would also only require a single localizer run. Specifically, instead of selecting and using a single (most-informative) channel for decoding participants’ intentions, a task-specific activation pattern would be defined after the localizer session (based on a univariate approach over each channel comprising the setup), here called as “*base-pattern.*” For each of the communication runs a new activation pattern for each condition would be calculated and compared to the *base-pattern* as in Monti et al. (2010). The answer option leading to the highest correlation or the smallest distance between the patterns would be the selected option. Importantly, the number of optodes comprising the setup should be optimized to guarantee participants’ comfort and a good accuracy level. In addition, due to the existing trial-to-trial variability within and across participants, a subject-specific number of trials could be considered instead of seeking a group-based criterion. This could be achieved, for example, by implementing an evidence accumulation process with a stopping criterion that trades speed and accuracy for each participant (Mattout et al., 2015).

### Feasibility of Error-Correction Approach

Automatic recognition of error potentials has been successfully used in EEG-based BCIs that focus on sensorimotor rhythms and event-related potentials, since evoked responses by the feedback differ depending on whether the feedback is correct or not (Chavarriaga et al., 2014; Mattout et al., 2015). Hemodynamic signals do not show such distinct patterns, which makes direct forms of error-correction mechanisms more challenging to implement. Here we developed an active error-correction approach where participants were asked to indicate a decoding error by encoding the “Error” option in the next choice run if the decoded choice they received did not correspond to what they intended to encode. This approach assumes that we can correctly detect the “Error” option when participants encode it. We built a confusion matrix by pooling all encoded answers across participants to evaluate the performance of our proposed error detection approach. In an ideal scenario, the number of “Error” trials comprising this matrix should be zero or close to zero, which would indicate that no decoding mistakes were made. The fact that participants reached an average of ∼74% accuracy indicates that participants had to encode “Error” several times, but importantly, this number differed across participants. Figure 7 (right side) shows that for example, P06 had to encode “Error” 5/8 times, while P02 did not have to encode any. The figure also indicates that the number of “Error” trials was lower than “not Error” trials (thus making the confusion matrix unbalanced). The confusion matrix shows that we reached an accuracy of 90.62% (72/96 trials were correctly labeled as “Error” or “not Error”). However, we only reached a recall level of 63.63% (out of 22 error trials, 8 trials were missed), which indicates that this approach did not always work.

It is also important to note that the number of encoded errors does not directly represent the accuracy of the BCI setup. This is due to three reasons: first of all, owing to a technical mistake, data from P01-P07 were reanalyzed offline. In turn, some trials that were incorrectly decoded in real-time were correctly decoded offline (and *vice versa*), which misplaced the presence of “Error” encoding runs (and disrupted the semantic link between the encoded and decoded choices). This means that in the former case (after offline analysis the choice was correctly decoded), a subsequent error-encoding run became unnecessary, while for the latter case (after offline analysis the choice run was incorrectly decoded) a following “Error” encoding run should have occurred (see Supplementary Data Sheet S1). Second, our experimental design did not include an error option in the first level of the nested menu. This implies that if choices were wrongly decoded in the fourth level of the menu, participants were no longer able to encode the “Error” option in the next run. Third, and similarly, if a decoding error occurred in the last run of the experiment (run number eight), participants were no longer able to encode the “Error” option. These two scenarios could be addressed in the future by using additional short runs (under a minute) where the participant would verify if the decoded answer was correct or not. The run would consist of an initial and final baseline periods of 20 and 10 s, respectively, with a single full rotation of the AR cube presented in between. Specifically, the AR cube would show faces corresponding to yes/no answers, alternated with rest periods, i.e., YES-NO-REST-YES-NO-REST (6 s per face, 36 s in total). This would allow participants to encode twice whether the decoding option was correct or not in 66 s, while leaving enough time for the hemodynamic response to get back to baseline.

In this experiment participants navigated through a four-level, nested menu. After completing one full round (i.e., reaching level four), participants were directed back to the first level of the menu. Since participants performed eight choice runs, this structure allowed them to maximally go through the menu twice. Due to the technical mistake mentioned above, the following lines will only discuss results pertaining P08–P12: P11 completed two full rounds (100% accuracy), while P09 and P10 completed one full round (both participants reached a 87.5% accuracy); P08 and P12 did not manage to complete a single round (the decoding accuracy for both participants was 50%). These results clearly show that *statistically* significant accuracy is a necessary but not sufficient prerequisite to achieve a *functionally* significant accuracy. Indeed, the accuracy that would be necessary to use the system in a convenient way requires the accuracy to be much higher. Future work should include the “Error” option in each level of the nested menu. It should also consider an additional measure besides the magnitude of the *t*-statistic for decoding participants’ choices, such as a confidence measure based on the absolute differences in the *t*-estimate across conditions. We expect that a more informed decision helps improving the decoding and the error-detection processes.

### Task Selection Based on Participants’ Preference and Previous Experience

In the present study, we first trained participants to perform two different motor imagery tasks and subsequently let them choose their preferred option. However, unlike previous work, we did not test whether user preference leads to better performance compared to an experimenter-based task selection approach (Weyand and Chau, 2017).

Intuitively, experienced BCI users may have a more realistic idea of which mental strategy works best for them and thus choose the task that has worked well in the past. Although we asked participants to choose the task they felt most comfortable with in the given setup independent of their previous experience, P02, P04, P05, and P11 chose to use mental drawing for this very reason. In contrast, participants P07 and P10, who also reported being familiar with the mental drawing task (and unfamiliar to the interacting with the cube task), chose to use interacting with the cube as it felt more natural for them given the AR stimuli.

Previous experience with the mental task, BCI setups and fNIRS systems did not show significant correlation with obtained accuracies. However, differences in decoding accuracies between (1) novices and (2) average and more experienced BCI/fNIRS users were considerably high [65.63% vs. 80% (average) and 75% (more experienced) for BCI and 62.5% vs. 70% (average) and 82.5% (more experienced) for fNIRS]. Similarly, we observed differences between the same groups but to a lower extent regarding previous task experience. Specifically, novices reached a mean accuracy of 71.73%, while average and more experienced users reached 75 and 79.17%, respectively. These observations suggest that participants with a certain level of experience with a BCI/fNIRS system or a given mental task may have enough introspective information to make an adequate and informed decision on their preferred task after a single training session.

### Using AR in BCIs Offers a Great Flexibility

Recent work has shown that EEG-based BCIs can successfully be used in combination with new technological developments such as AR to improve real-world practicality by offering a richer, more direct, and intuitive interface (Kansaku et al., 2010; Takano et al., 2011; Borges et al., 2016; Faller et al., 2017). However, very few fNIRS applications have explored this option (Afergan et al., 2015; McKendrick et al., 2016; Si-Mohammed et al., 2018; Hu et al., 2019). In the present study, we employed an AR cube to guide the temporal-encoding approach and to display the decoded answer of participants’ intentions. For that, we used a relatively simple and flexible setup from the hardware point of view: we made use of two laptops, one additional computer screen, an HD webcam, and home-made A4 cardboards. The home-made A4 cardboards served as whiteboards and triggered the display of the AR cube in Unity3D on an additional computer screen. Importantly, a whiteboard offers a high degree of flexibility and individuality as anyone (a caretaker, family member, experimenter, etc.) could write potential choice options based on previous knowledge of the user and/or the social context (although we used the same choice options for all participants in this experiment, see Supplementary Data Sheet S2). Also, a whiteboard provides a degree of proximity to the setup and interaction between the user and the experimenter as new choice options need to be written down after each run. Besides, handwriting may offer a sense of familiarity to the user. It is important to note that participants were instructed to look at the computer screen at all times throughout the runs, which makes the chosen location of the cardboard (on the desk, between the computer screen and the participant) not intuitive from a pure AR setup perspective. Indeed, the cardboard could have been placed in a different location (behind the screen, for example) as long as the webcam’s placement would change accordingly. However, we chose consciously to place the cardboard between the screen and the participants exclusively to exploit the cardboards’ interactive and proximity features mentioned above.

Altogether, this relatively simple setup has the potential to be successfully implemented in a more ecologically valid environment such as a hospital room or a rehab center. From the setup point of view, we picture a situation where the user would be placed comfortably in a Fowler’s position (head is placed at a 45-degree angle), while wearing the optodes, fNIRS cap and overcap. The fNIRS system would be located next to the bed. A removable desk would be attached to the structure of the bed, above the user’s thighs, slightly tilted toward the user’s head. A tablet fixated almost perpendicular to the desk could be used instead of the additional computer screen to display the AR cube. To maximize comfort, the rotation of the desk would be adjusted to ensure the tablet was placed at the same height as the user’s eye gaze. The webcam would be integrated into the tablet or a separate camera would be placed on a stable structure such as a tripod located right next to the participant and it would be recording the contents of the whiteboard. Alternatively, smart glasses with an integrated camera could be used. These glasses would then also replace the tablet and could display the cube directly on the glasses.

From the data analysis point of view, the current decoding process could be improved to increase the performance of the BCI (as discussed in previous sections). Importantly, as the majority of the analysis steps have been streamlined (through scripts written in Matlab and Unity3D), a single BCI operator would be sufficient to perform the measurements. However, to assure that the channel selection procedure is properly done (i.e., the selected channel is sufficiently informative and not corrupted extensively by noise), an experienced researcher or a trained medical professional in understanding the fNIRS signal would be necessary. Of course, caretakers and family members should be encouraged at all times to assist the experimenter in selecting the most appropriate options to be presented to the user through the whiteboard.

## Conclusion and Outlook

In the present study, we showed that fNIRS-based BCIs can be successfully combined with AR technology to address a six-class problem using a single mental task and fNIRS channel. AR technology allows for a seamless real-world interaction that future studies should explore in more detail. The high inter-subject variability observed in this study not only in achieved accuracies but also in task preference and channel selection, points at the need of shifting the BCI field toward a true user-centered approach. Future studies should consider pursuing individualized approaches to bridge the gap from research to real-world applications.

## Data Availability Statement

The raw data supporting the conclusions of this article will be made available by the authors, without undue reservation, to any qualified researcher.

## Ethics Statement

The studies involving human participants were reviewed and approved by ethics committee of the Faculty of Psychology and Neuroscience, Maastricht University, The Netherlands. The patients/participants provided their written informed consent to participate in this study.

## Author Contributions

RBu, AB-A, and BS conceived the idea and designed the experiment. RBe and RBu prepared the Unity3D environment. RBe developed the dynamic object control functions. RBu, ML, and AB-A optimized the hardware setup. RBu and AB-A measured pilot participants. BS and AB-A adapted the design based on pilot measurements. AB-A carried out the fNIRS measurements, analyzed the data, and wrote the manuscript. BS, ML, and RM assisted on the interpretation of results and data analysis. AB-A, BS, and ML structured the manuscript. RBu, BS, RM, ML, RM, and RBe revised the manuscript critically.

## Conflict of Interest

The authors declare that the research was conducted in the absence of any commercial or financial relationships that could be construed as a potential conflict of interest.

## Supplementary Material

DATA SHEET S1 Encoded and decoded choices.

DATA SHEET S2 Menu choices presented to participants.
