# Supplementary material for: An Augmented-Reality fNIRS-Based Brain-Computer Interface: A Proof-of-Concept Study
Source: Front Neurosci. 2020 Apr 28;14:346. doi: 10.3389/fnins.2020.00346 (PMC7199634; doi:10.3389/fnins.2020.00346)
Supplement: DATA SHEET S1 — Encoded and decoded choices. [file Data_Sheet_1.pdf]

Encoded and decoded choices

This document compiles all encoded (by participants) and decoded choices (based on the temporal decoding approach) for each participant. It is important to note that, the data from P01 to P07 were reanalyzed offline due to a technical mistake. Thus, some trials that were incorrectly decoded in real-time were correctly decoded offline (and vice versa), which misplaced the presence of "Error" encoding runs (and disrupted the semantic link between the encoded and decoded choices). This is clearly observed in participants P03 (Table 3) to P07 (Table 7). Choices in **green** indicate a *successful* completion of the (four-level) navigation round, while choices in **red** indicate an *unsuccessful* completion of the navigation round.

Table 1. Encoded and decoded choices for P01

	**Encoded** *(based on real-time experiment)*		**Decoded** *(based on offline analysis)*	
	Name	*choice #*	*Name*	*choice #*
**Run 1**	Music	1	Photo	4
**Run 2**	Error	1	Error	1
**Run 3**	Music	1	Music	1
**Run 4**	Jazz	3	Classical	1
**Run 5**	Error	2	Error	2
**Run 6**	Jazz	3	Jazz	3
**Run 7**	Nina Simone	4	Nina Simone	4
**Run 8**	I put a spell on you	1	**I put a spell on you**	1

Note: due to a technical mistake, the choice order of Jazz artists presented to P01 was different than for the rest of the participants

Table 2. Encoded and decoded choices for P02

	**Encoded** *(based on real-time experiment)*		**Decoded** *(based on offline analysis)*	
	Name	*choice #*	*Name*	*choice #*
**Run 1**	Photo	4	Photo	4
**Run 2**	Pets	6	Pets	6
**Run 3**	Album 1	3	Album 1	3
**Run 4**	Picture 5	1	**Picture 5**	1
**Run 5**	Other	6	Other	6
**Run 6**	Room Control	1	Room Control	1
**Run 7**	Bed	6	Bed	6
**Run 8**	Move Up	2	**Move Up**	2

Table 3. Encoded and decoded choices for P03

	**Encoded** *(based on real-time experiment)*		**Decoded** *(based on offline analysis)*	
	Name	*choice #*	*Name*	*choice #*
**Run 1**	TV	2	TV	2
**Run 2**	Movie	1	Movie	1
**Run 3**	Action	4	Error	1
**Run 4**	Error	4	Series/News	4
**Run 5**	Action	4	Game Show	4
**Run 6**	Terminator	5	**First Date**	5
**Run 7**	Read	3	Other	6
**Run 8**	Error	2	Internet	5

Table 4. Encoded and decoded choices for P04

	**Encoded** *(based on real-time experiment)*		**Decoded** *(based on offline analysis)*	
	Name	*choice #*	*Name*	*choice #*
**Run 1**	TV	2	TV	2
**Run 2**	Series/News	4	Movies	1
**Run 3**	Error	1	Sci-Fi	2
**Run 4**	Series/News	4	**The martian**	5
**Run 5**	Comedy	3	Read	3
**Run 6**	The good place	6	Comic	1
**Run 7**	Music	1	Superhero	1
**Run 8**	Rock	2	**Capt. America**	5

Table 5. Encoded and decoded choices for P05

	Encoded (based on real-time experiment)		Decoded (based on offline analysis)	
	Name	*choice #*	*Name*	*choice #*
**Run 1**	Photo	4	Photo	4
**Run 2**	Error	6	Error	1
**Run 3**	Error	2	Music	1
**Run 4**	Albeniz	1	Classical	1
**Run 5**	Cordoba	3	Mozart	3
**Run 6**	TV	2	**Magic Flute**	2
**Run 7**	Series	4	Photo	4
**Run 8**	Children	6	Pets	6

Table 6. Encoded and decoded choices for P06

	Encoded (based on real-time experiment)		Decoded (based on offline analysis)	
	Name	*choice #*	*Name*	*choice #*
**Run 1**	Music	1	Music	1
**Run 2**	Hip-Hop	5	Hip-hop	5
**Run 3**	Error	2	Error	2
**Run 4**	Error	5	Hip-hop	5
**Run 5**	Error	2	Jay-Z	3
**Run 6**	Error	5	**King´s Dead**	6
**Run 7**	Error	2	TV	2
**Run 8**	Hip-Hop	5	Documentaries	2

Table 7. Encoded and decoded choices for P07

	**Encoded** *(based on real-time experiment)*		**Decoded** *(based on offline analysis)*	
	Name	*choice #*	*Name*	*choice #*
**Run 1**	Radio	5	Radio	5
**Run 2**	Spain	2	Spain	2
**Run 3**	Error	4	Error	4
**Run 4**	Error	6	Germany	6
**Run 5**	Music	1	**FM1**	1
**Run 6**	Rock	2	Program 5	5
**Run 7**	Error	2	TV	2
**Run 8**	Error	5	Shows/News	5

Table 8. Encoded and decoded choices for P08

	**Encoded**		**Decoded**	
	Name	*choice #*	*Name*	*choice #*
**Run 1**	Music	1	Music	1
**Run 2**	Jazz	3	Jazz	3
**Run 3**	Louis Armstrong	4	Louis Armstrong	4
**Run 4**	Let´s do it	3	**Blue Skies**	1
**Run 5**	Read	3	Read	3
**Run 6**	Magazine	5	Poetry	3
**Run 7**	Error	5	Emily Dickinson	3
**Run 8**	Error	3	Error	1

Table 9. Encoded and decoded choices for P09

	**Encoded**		**Decoded**	
	Name	*choice #*	*Name*	*choice #*
**Run 1**	TV	2	Radio	5
**Run 2**	Error	5	Error	5
**Run 3**	TV	2	TV	2
**Run 4**	Movies	1	Movies	1
**Run 5**	Sci-Fi	2	Sci-Fi	2
**Run 6**	Star Wars	3	**Star Wars**	3
**Run 7**	Photo	4	Photo	4
**Run 8**	Pets	6	Pets	6

Table 10. Encoded and decoded choices for P10

	Encoded		Decoded	
	Name	*choice #*	*Name*	*choice #*
**Run 1**	Music	1	Music	1
**Run 2**	Classical	1	Classical	1
**Run 3**	Chopin	6	Chopin	6
**Run 4**	Requiem	1	**Requiem**	1
**Run 5**	TV	2	TV	2
**Run 6**	Movies	1	Movies	1
**Run 7**	Sci-Fi	2	Sci-Fi	2
**Run 8**	The martian	5	Interstellar	1

Table 11. Encoded and decoded choices for P11

	Encoded		Decoded	
	Name	*choice #*	*Name*	*choice #*
**Run 1**	Photo	4	Photo	4
**Run 2**	Pets	6	Pets	6
**Run 3**	Album 2	2	Album 2	2
**Run 4**	Picture 1	1	**Picture 1**	1
**Run 5**	Read	3	Read	3
**Run 6**	Poetry	3	Poetry	3
**Run 7**	Pablo Neruda	1	Pablo Neruda	1
**Run 8**	Die Slowly	5	**Die Slowly**	5

Table 12. Encoded and decoded choices for P12

	Encoded		Decoded	
	Name	*choice #*	*Name*	*choice #*
**Run 1**	Photo	4	Other	6
**Run 2**	Error	2	Error	2
**Run 3**	Photo	4	Music	1
**Run 4**	Error	6	Error	6
**Run 5**	Photo	4	Photo	4
**Run 6**	Pets	6	Pets	6
**Run 7**	Album 3	3	Error	6
**Run 8**	Error	1	Trips I	2
