# Supplementary material for: An Augmented-Reality fNIRS-Based Brain-Computer Interface: A Proof-of-Concept Study
Source: Front Neurosci. 2020 Apr 28;14:346. doi: 10.3389/fnins.2020.00346 (PMC7199634; doi:10.3389/fnins.2020.00346)
Supplement: DATA SHEET S2 — Menu choices presented to participants. [file Data_Sheet_2.pdf]

Menu choices presented to participants

Schematic representation of the procedure

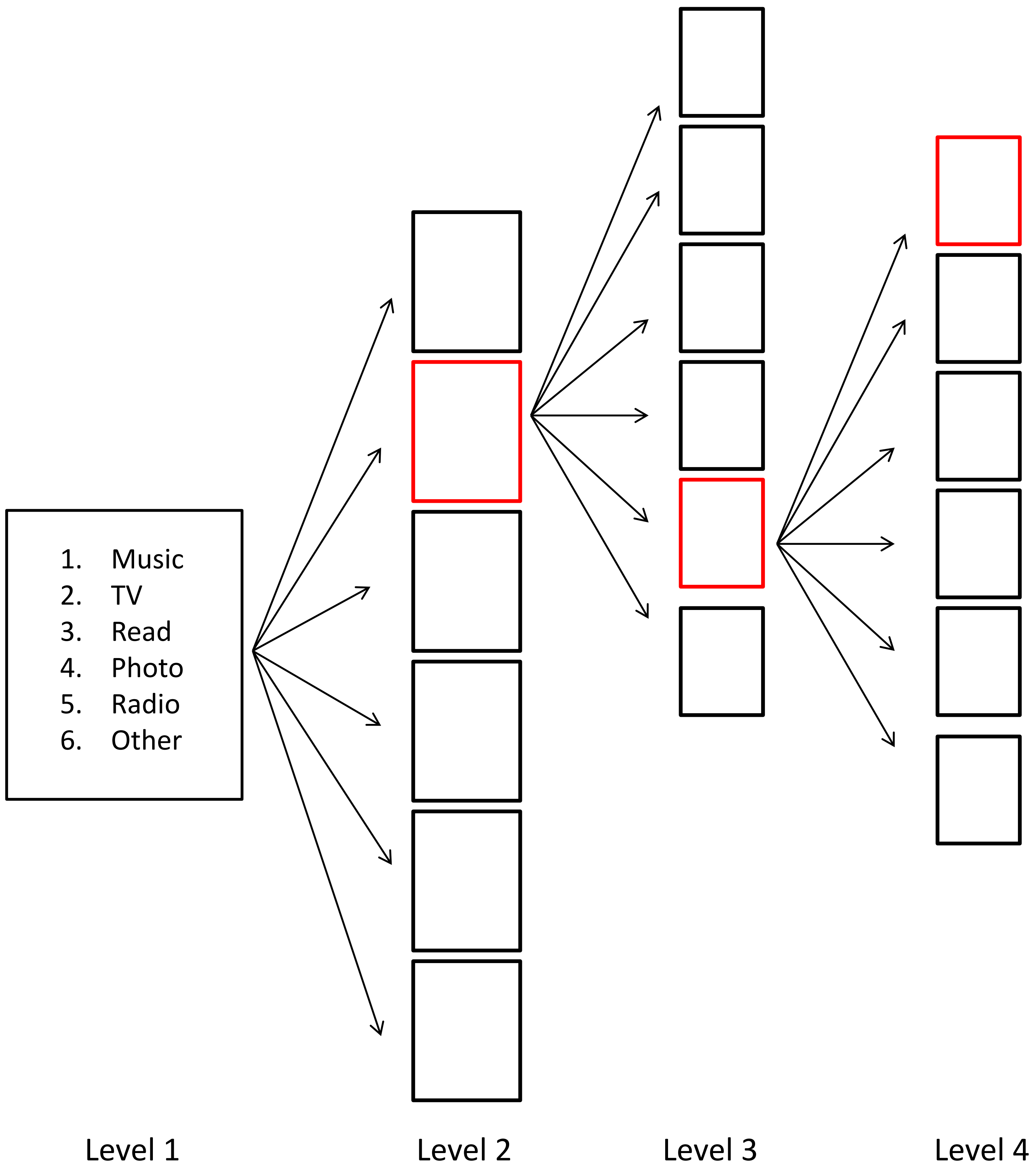

MUSIC

Level 1

1. Music
2. TV
3. Read
4. Photo
5. Radio
6. Other

Level 2

1. Classical
2. Rock
3. Jazz
4. Pop
5. Hip-hop
6. Error

Level 3

1. Albeniz
2. Error
3. Mozart
4. Beethoven
5. Vivaldi
6. Chopin

1. Albeniz
2. Error
3. Mozart
4. Beethoven
5. Vivaldi
6. Chopin

1. Albeniz
2. Error
3. Mozart
4. Beethoven
5. Vivaldi
6. Chopin

1. Albeniz
2. Error
3. Mozart
4. Beethoven
5. Vivaldi
6. Chopin

1. Albeniz
2. Error
3. Mozart
4. Beethoven
5. Vivaldi
6. Chopin

Level 4

1. Granada
2. Asturias
3. Cordoba
4. Oriental
5. Error
6. Azulejos

1. Requiem
2. Magic Flute
3. Haydn Quartets
4. Clarinet Concerto
5. Error
6. Jupiter

1. Moonlight s.
2. Simphony #7
3. Adelaide
4. Fur Elise
5. Error
6. Fidelio

1. Gloria
2. Stabat Mater
3. L’Olimpiade
4. Bajazet
5. Error
6. Four seasons

1. Raindrop
2. Nocturne
3. Romance
4. Cello s.
5. Error
6. Tristesse

1. Music
2. TV
3. Read
4. Photo
5. Radio
6. Other

1. Classical
2. Rock
3. Jazz
4. Pop
5. Hip-hop
6. Error

1. Led Zeppelin
2. Error
3. The Beatles
4. Rolling Stones
5. Queen
6. Radiohead

1. Kashmir
2. Ramble on
3. Stairway to heaven
4. Black dog
5. Error
6. The Ocean

1. Led Zeppelin
2. Error
3. The Beatles
4. Rolling Stones
5. Queen
6. Radiohead

1. Hey Jude
2. Let it be
3. Yesterday
4. Love me do
5. Error
6. Twist & Shout

1. Led Zeppelin
2. Error
3. The Beatles
4. Rolling Stones
5. Queen
6. Radiohead

1. Satisfaction
2. Paint It, Black
3. Sway
4. Shine A Light
5. Error
6. Gimme Shelter

1. Led Zeppelin
2. Error
3. The Beatles
4. Rolling Stones
5. Queen
6. Radiohead

1. Bohemian rhapsody
2. Don’t stop me now
3. Radio Ga Ga
4. I want to break free
5. Error
6. Under Pressure

1. Led Zeppelin
2. Error
3. The Beatles
4. Rolling Stones
5. Queen
6. Radiohead

1. No surprises
2. Exit music
3. Karma police
4. Creep
5. Error
6. Nude

Presented to P01 (technical mistake)

1. Music
2. TV
3. Read
4. Photo
5. Radio
6. Other

1. Classical
2. Rock
3. Jazz
4. Pop
5. Hip-hop
6. Error

1. Ella Fitzgerald
2. Bill Evans
3. Louis Armstrong
4. Nina Simone
5. Julian Lage
6. Error

1. Blue skies
2. Misty
3. Let's do it
4. Oh lady be good
5. Error
6. Summertime

1. Ella Fitzgerald
2. Bill Evans
3. Louis Armstrong
4. Nina Simone
5. Julian Lage
6. Error

1. But Beautiful
2. Autumn Leaves
3. My Foolish Heart
4. Reflections in D
5. Error
6. Never Let Me Go

1. Ella Fitzgerald
2. Bill Evans
3. Louis Armstrong
4. Nina Simone
5. Julian Lage
6. Error

1. Jeepers Creepers
2. Hello Dolly
3. You'll never walk alone
4. Ain't misbehaving
5. Error
6. Georgia on my mind

1. Ella Fitzgerald
2. Bill Evans
3. Louis Armstrong
4. Nina Simone
5. Julian Lage
6. Error

1. I put a spell on you
2. Four women
3. Sinnerman
4. Baltimore
5. Error
6. Feeling Good

1. Ella Fitzgerald
2. Bill Evans
3. Louis Armstrong
4. Nina Simone
5. Julian Lage
6. Error

1. Persian rug
2. Crying
3. In heaven
4. Harlem Blues
5. Error
6. Nocturne

Presented to P02-P12

1. Music
2. TV
3. Read
4. Photo
5. Radio
6. Other

1. Classical
2. Rock
3. Jazz
4. Pop
5. Hip-hop
6. Error

1. Ella Fitzgerald
2. Error
3. Bill Evans
4. Louis Armstrong
5. Nina Simone
6. Julian Lage

1. Blue skies
2. Misty
3. Let's do it
4. Oh lady be good
5. Error
6. Summertime

1. Ella Fitzgerald
2. Error
3. Bill Evans
4. Louis Armstrong
5. Nina Simone
6. Julian Lage

1. But Beautiful
2. Autumn Leaves
3. My Foolish Heart
4. Reflections in D
5. Error
6. Never Let Me Go

1. Ella Fitzgerald
2. Error
3. Bill Evans
4. Louis Armstrong
5. Nina Simone
6. Julian Lage

1. Jeepers Creepers
2. Hello Dolly
3. You'll never walk alone
4. Ain't misbehaving
5. Error
6. Georgia on my mind

1. Ella Fitzgerald
2. Error
3. Bill Evans
4. Louis Armstrong
5. Nina Simone
6. Julian Lage

1. I put a spell on you
2. Four women
3. Sinnerman
4. Baltimore
5. Error
6. Feeling Good

1. Ella Fitzgerald
2. Error
3. Bill Evans
4. Louis Armstrong
5. Nina Simone
6. Julian Lage

1. Persian rug
2. Crying
3. In heaven
4. Harlem Blues
5. Error
6. Nocturne

1. Music
2. TV
3. Read
4. Photo
5. Radio
6. Other

1. Classical
2. Rock
3. Jazz
4. Pop
5. Hip-hop
6. Error

1. Pink
2. Error
3. Beyoncé
4. Justin Bieber
5. Ariana Grande
6. Michael Jackson

1. Try
2. Raise your glass
3. So what
4. Who knew
5. Error
6. Blow me

1. Pink
2. Error
3. Beyoncé
4. Justin Bieber
5. Ariana Grande
6. Michael Jackson

1. Baby boy
2. Crazy in love
3. Drunk in love
4. Single ladies
5. Error
6. Halo

1. Pink
2. Error
3. Beyoncé
4. Justin Bieber
5. Ariana Grande
6. Michael Jackson

1. Love me
2. What do you mean?
3. Boyfriend
4. Friends
5. Error
6. Baby

1. Pink
2. Error
3. Beyoncé
4. Justin Bieber
5. Ariana Grande
6. Michael Jackson

1. Bang bang
2. Side to side
3. Problem
4. Into you
5. Error
6. In my head

1. Pink
2. Error
3. Beyoncé
4. Justin Bieber
5. Ariana Grande
6. Michael Jackson

1. Billy Jean
2. Thriller
3. Beat it
4. Man in the mirror
5. Error
6. Smooth criminal

1. Music
2. TV
3. Read
4. Photo
5. Radio
6. Other

1. Classical
2. Rock
3. Jazz
4. Pop
5. Hip-hop
6. Error

1. Kendrick Lamar
2. Error
3. Jay-Z
4. Eminem
5. 50 cent
6. Kanye West

1. Money trees
2. Humble
3. Loyalty
4. DNA
5. Error
6. King's dead

1. Kendrick Lamar
2. Error
3. Jay-Z
4. Eminem
5. 50 cent
6. Kanye West

1. Run this town
2. 99 problems
3. Anything
4. Empire State of Mind
5. Error
6. Izzo

1. Kendrick Lamar
2. Error
3. Jay-Z
4. Eminem
5. 50 cent
6. Kanye West

1. 8 miles
2. Without me
3. Till I collapse
4. Lose yourself
5. Error
6. No love

1. Kendrick Lamar
2. Error
3. Jay-Z
4. Eminem
5. 50 cent
6. Kanye West

1. In da house
2. Just a lil bit
3. Wanksta
4. Window shopper
5. Error
6. Candy shop

1. Kendrick Lamar
2. Error
3. Jay-Z
4. Eminem
5. 50 cent
6. Kanye West

1. Flashing lights
2. Mercy
3. Gold digger
4. Stronger
5. Error
6. Power

TV

1. Music
2. TV
3. Read
4. Photo
5. Radio
6. Other

Level 1

1. Movies
2. Documentaries
3. Error
4. Series/news
5. Shows
6. Sports

Level 2

1. Error
2. Sci-Fi
3. Drama
4. Action
5. Horror
6. Romance

1. Interstellar
2. The Matrix
3. Star Wars
4. Error
5. The martian
6. Ex machina

1. Error
2. Sci-Fi
3. Drama
4. Action
5. Horror
6. Romance

1. Spotlight
2. Carol
3. Boyhood
4. Error
5. Moonlight
6. The King's speech

1. Error
2. Sci-Fi
3. Drama
4. Action
5. Horror
6. Romance

1. Fast & Furious
2. The Dark Knight
3. Mad Max
4. Error
5. Terminator
6. Casino Royale

1. Error
2. Sci-Fi
3. Drama
4. Action
5. Horror
6. Romance

1. Saw
2. It
3. Jaw
4. Error
5. Psycho
6. The exhorcist

1. Error
2. Sci-Fi
3. Drama
4. Action
5. Horror
6. Romance

1. The notebook
2. Titanic
3. Dear John
4. Error
5. Love actually
6. Casablanca

Level 3

Level 4

1. Music
2. TV
3. Read
4. Photo
5. Radio
6. Other

1. Movies
2. Documentaries
3. Error
4. Series/news
5. Shows
6. Sports

1. Error
2. Nature
3. Biography
4. History
5. Science
6. Travelling

1. Blackfish
2. Planet Earth
3. The ivory game
4. Error
5. Our planet
6. Chasing ice

1. Error
2. Nature
3. Biography
4. History
5. Science
6. Travelling

1. RBG
2. Mercury 13
3. Pumping iron
4. Error
5. Amy
6. Searching for sugarman

1. Error
2. Nature
3. Biography
4. History
5. Science
6. Travelling

1. Power's war
2. The great plague
3. Above & beyond
4. Error
5. The civil war
6. The thin blue line

1. Error
2. Nature
3. Biography
4. History
5. Science
6. Travelling

1. Particle fever
2. Dinosaur 13
3. Resistance
4. Error
5. Icarus
6. Cosmos

1. Error
2. Nature
3. Biography
4. History
5. Science
6. Travelling

1. 180 south
2. A map for Saturday
3. Baraka
4. Error
5. The maiden trip
6. Pedal the world

1. Music
2. TV
3. Read
4. Photo
5. Radio
6. Other

1. Movies
2. Documentaries
3. Error
4. Series/news
5. Shows
6. Sports

1. Error
2. News
3. Comedy
4. Cooking
5. Sci-Fi/Fantasy
6. Children

1. BBC
2. CNN
3. Euronews
4. Error
5. Al Jazeera
6. Geonews

1. Error
2. News
3. Comedy
4. Cooking
5. Sci-Fi/Fantasy
6. Children

1. Friends
2. It's always sunny
3. How I met your mother
4. Error
5. The Big Bang Theory
6. The good place

1. Error
2. News
3. Comedy
4. Cooking
5. Sci-Fi/Fantasy
6. Children

1. Ugly delicious
2. Chef's table
3. No reservations
4. Error
5. Hell's kitchen
6. Parts unknown

1. Error
2. News
3. Comedy
4. Cooking
5. Sci-Fi/Fantasy
6. Children

1. Black mirror
2. Game of thrones
3. Altered carbon
4. Error
5. Sense 8
6. Travellers

1. Error
2. News
3. Comedy
4. Cooking
5. Sci-Fi/Fantasy
6. Children

1. Peppa pig
2. Sponge bob
3. Sesame street
4. Error
5. PAW patrol
6. Scooby doo

1. Music
2. TV
3. Read
4. Photo
5. Radio
6. Other

1. Movies
2. Documentaries
3. Error
4. Series/news
5. Shows
6. Sports

1. Error
2. Dating show
3. Home Shopping
4. Game show
5. Talk show
6. Reality show

1. Dismissed
2. Love island
3. Date my mom
4. Error
5. First date hotel
6. Splits villa

1. Error
2. Dating show
3. Home Shopping
4. Game show
5. Talk show
6. Reality show

1. TV-winkel
2. QVC
3. HSN
4. Error
5. Shop TV
6. ShopNBC

1. Error
2. Dating show
3. Home Shopping
4. Game show
5. Talk show
6. Reality show

1. Who wants to be a $$
2. Minute to win it
3. Takeshi's castle
4. Error
5. Guess the prize
6. Family feud

1. Error
2. Dating show
3. Home Shopping
4. Game show
5. Talk show
6. Reality show

1. Last week tonight
2. Ellen's show
3. Oprah's show
4. Error
5. Daily show
6. David Letterman

1. Error
2. Dating show
3. Home Shopping
4. Game show
5. Talk show
6. Reality show

1. Queer eye
2. Survivor
3. The Colony
4. Error
5. Big brother
6. Jersey shore

1. Music
2. TV
3. Read
4. Photo
5. Radio
6. Other

1. Movies
2. Documentaries
3. Error
4. Series/news
5. Shows
6. Sports

1. Error
2. Eurosport
3. Golf channel
4. Sky sports
5. Premier sports
6. Fox sports

1. Wimbledon
2. Nations cup
3. World cup
4. Error
5. Giro
6. Tour de France

1. Error
2. Eurosport
3. Golf channel
4. Sky sports
5. Premier sports
6. Fox sports

1. Golf central
2. European tour
3. PGA tour
4. Error
5. Ladies tour
6. Playing lessons

1. Error
2. Eurosport
3. Golf channel
4. Sky sports
5. Premier sports
6. Fox sports

1. Best premier league
2. Sports news
3. Football: greatest of
4. Error
5. US Open
6. Good morning fans

1. Error
2. Eurosport
3. Golf channel
4. Sky sports
5. Premier sports
6. Fox sports

1. Copa america
2. NHL
3. Nacas america
4. Error
5. CSL
6. Concacaf

1. Error
2. Eurosport
3. Golf channel
4. Sky sports
5. Premier sports
6. Fox sports

1. Motocross
2. US senior open
3. Formula 1
4. Error
5. Moto GP
6. Triathlon

Read

1. Music
2. TV
3. **Read**
4. Photo
5. Radio
6. Other

Level 1

1. **Comic**
2. Novel
3. Poetry
4. Error
5. Magazine
6. Newspaper

Level 2

1. **Superhero**
2. Gag-a-day
3. Romance
4. Crime
5. Error
6. Horror

1. Batman
2. Superman
3. Error
4. Wonder Woman
5. Cpt. America
6. Green Lantern

1. Superhero
2. **Gag-a-day**
3. Romance
4. Crime
5. Error
6. Horror

1. The awkward Yeti
2. Garfield
3. Error
4. Calvin & Hobbes
5. Snoopy & Peanuts
6. Dilbert

1. Superhero
2. Gag-a-day
3. **Romance**
4. Crime
5. Error
6. Horror

1. Drowning girl
2. In the car
3. Error
4. Hopeless
5. In the car
6. Secret hearts

1. Superhero
2. Gag-a-day
3. Romance
4. **Crime**
5. Error
6. Horror

1. Criminal
2. A dame to kill for
3. Error
4. Sin city
5. Scalped
6. From hell

1. Superhero
2. Gag-a-day
3. Romance
4. Crime
5. Error
6. **Horror**

1. The haunt of fear
2. The vault of horror
3. Error
4. Tales from the crypt
5. Tomb of terror
6. The thing

Level 3

Level 4

1. Music
2. TV
3. Read
4. Photo
5. Radio
6. Other

1. Comic
2. Novel
3. Poetry
4. Error
5. Magazine
6. Newspaper

1. Sci-fi
2. Romance
3. Dystopia
4. Mystery
5. Error
6. Historical

1. Frankenstein
2. Neuromancer
3. Error
4. Lord of the Rings
5. Ender’s game
6. The hunger games

1. Sci-fi
2. Romance
3. Dystopia
4. Mystery
5. Error
6. Historical

1. Jane Eyre
2. The notebook
3. Error
4. Me before you
5. Outlander
6. This man

1. Sci-fi
2. Romance
3. Dystopia
4. Mystery
5. Error
6. Historical

1. The handmaid’s tail
2. 1984
3. Error
4. A brave mew world
5. Dune
6. The giver

1. Sci-fi
2. Romance
3. Dystopia
4. Mystery
5. Error
6. Historical

1. Rebecca
2. Gone girl
3. Error
4. The Firm
5. Eye of the needle
6. The alinenist

1. Sci-fi
2. Romance
3. Dystopia
4. Mystery
5. Error
6. Historical

1. War and peace
2. The pillars of the earth
3. Error
4. Wolf hall
5. Gone with the wind
6. The English patient

1. Music
2. TV
3. Read
4. Photo
5. Radio
6. Other

1. Comic
2. Novel
3. Poetry
4. Error
5. Magazine
6. Newspaper

1. Pablo Neruda
2. Maya Angelou
3. Emily Dickinson
4. Robert Frost
5. Error
6. Whitman

1. Your hands
2. If you forget me
3. Error
4. Your laughter
5. Die slowly
6. Here I love you

1. Pablo Neruda
2. Maya Angelou
3. Emily Dickinson
4. Robert Frost
5. Error
6. Whitman

1. The traveller
2. Glory falls
3. Error
4. Son to mother
5. Equality
6. When you come

1. Pablo Neruda
2. Maya Angelou
3. Emily Dickinson
4. Robert Frost
5. Error
6. Whitman

1. A doubt if it be us
2. A clock stopped
3. Error
4. All that I do
5. Besides this May
6. Experiment to me

1. Pablo Neruda
2. Maya Angelou
3. Emily Dickinson
4. Robert Frost
5. Error
6. Whitman

1. Bond and Free
2. Birches
3. Error
4. The pastrue
5. Blueberries
6. Going for water

1. Pablo Neruda
2. Maya Angelou
3. Emily Dickinson
4. Robert Frost
5. Error
6. Whitman

1. This compost
2. To think of time
3. Error
4. Continuities
5. Sounds of the winter
6. Thoughts

1. Music
2. TV
3. Read
4. Photo
5. Radio
6. Other

1. Comic
2. Novel
3. Poetry
4. Error
5. Magazine
6. Newspaper

1. Architecture
2. Photography
3. Fashion
4. Science
5. Error
6. Wildlife

1. Dezeen
2. DesignBloom
3. Error
4. Architecture lab
5. Volume Zero
6. Nytt Room

1. Architecture
2. Photography
3. Fashion
4. Science
5. Error
6. Wildlife

1. Aperture
2. B&W
3. Error
4. Click
5. Foam
6. Rotor drone

1. Architecture
2. Photography
3. Fashion
4. Science
5. Error
6. Wildlife

1. Vogue
2. Marie Claire
3. Error
4. Glamour
5. Elle
6. Cosmopolitan

1. Architecture
2. Photography
3. Fashion
4. Science
5. Error
6. Wildlife

1. New scientist
2. Cosmos
3. Error
4. WIRED
5. Skeptic
6. Knowledge

1. Architecture
2. Photography
3. Fashion
4. Science
5. Error
6. Wildlife

1. Birdwatch
2. National Wildlife
3. Error
4. BBC wildlife
5. Birds & Blooms
6. The zoologist

1. Music
2. TV
3. Read
4. Photo
5. Radio
6. Other

1. Comic
2. Novel
3. Poetry
4. Error
5. Magazine
6. Newspaper

1. German
2. Dutch
3. American
4. Spanish
5. Error
6. French

1. Die Zeit
2. Bild
3. Error
4. Die Welt
5. Tagesspiegel
6. Handelsblatt

1. German
2. Dutch
3. American
4. Spanish
5. Error
6. French

1. De Telegraaf
2. De Limburger
3. Error
4. Metro
5. De Volkskrant
6. Trow

1. German
2. Dutch
3. American
4. Spanish
5. Error
6. French

1. The washington post
2. The Boston Globe
3. Error
4. The New York Times
5. USA today
6. The State

1. German
2. Dutch
3. American
4. Spanish
5. Error
6. French

1. El Pais
2. ABC
3. Error
4. Marca
5. El Diario Vasco
6. El Mundo

1. German
2. Dutch
3. American
4. Spanish
5. Error
6. French

1. Le Monde
2. Le Figaro
3. Error
4. Le Parisien
5. L'Humanite
6. Les Echos

Photo

Level 1

1. Music
2. TV
3. Read
4. Photo
5. Radio
6. Other

Level 2

1. Error
2. Trips I
3. Trips II
4. Events
5. Family & friends
6. Pets

Level 3

1. Cuba
2. Martinica
3. South Africa
4. Indonesia
5. Spain
6. Error

Level 4

1. Picture 1
2. Error
3. Picture 2
4. Picture 3
5. Picture 4
6. Picture 5

Level 3

1. Cuba
2. Martinica
3. South Africa
4. Indonesia
5. Spain
6. Error

Level 4

1. Picture 1
2. Error
3. Picture 2
4. Picture 3
5. Picture 4
6. Picture 5

Level 3

1. Cuba
2. Martinica
3. South Africa
4. Indonesia
5. Spain
6. Error

Level 4

1. Picture 1
2. Error
3. Picture 2
4. Picture 3
5. Picture 4
6. Picture 5

Level 3

1. Cuba
2. Martinica
3. South Africa
4. Indonesia
5. Spain
6. Error

Level 4

1. Picture 1
2. Error
3. Picture 2
4. Picture 3
5. Picture 4
6. Picture 5

Level 3

1. Cuba
2. Martinica
3. South Africa
4. Indonesia
5. Spain
6. Error

Level 4

1. Picture 1
2. Error
3. Picture 2
4. Picture 3
5. Picture 4
6. Picture 5

1. Music
2. TV
3. Read
4. Photo
5. Radio
6. Other

1. Error
2. Trips I
3. Trips II
4. Events
5. Family & friends
6. Pets

1. India
2. France
3. Slovenia
4. Sweden
5. Iceland
6. Error

1. Picture 1
2. Error
3. Picture 2
4. Picture 3
5. Picture 4
6. Picture 5

1. India
2. France
3. Slovenia
4. Sweden
5. Iceland
6. Error

1. Picture 1
2. Error
3. Picture 2
4. Picture 3
5. Picture 4
6. Picture 5

1. India
2. France
3. Slovenia
4. Sweden
5. Iceland
6. Error

1. Picture 1
2. Error
3. Picture 2
4. Picture 3
5. Picture 4
6. Picture 5

1. India
2. France
3. Slovenia
4. Sweden
5. Iceland
6. Error

1. Picture 1
2. Error
3. Picture 2
4. Picture 3
5. Picture 4
6. Picture 5

1. India
2. France
3. Slovenia
4. Sweden
5. Iceland
6. Error

1. Picture 1
2. Error
3. Picture 2
4. Picture 3
5. Picture 4
6. Picture 5

1. Music
2. TV
3. Read
4. Photo
5. Radio
6. Other

1. Error
2. Trips I
3. Trips II
4. Events
5. Family & friends
6. Pets

1. Wedding
2. Birthday
3. Christmas
4. Easter
5. Graduation
6. Error

1. Picture 1
2. Error
3. Picture 2
4. Picture 3
5. Picture 4
6. Picture 5

1. Wedding
2. Birthday
3. Christmas
4. Easter
5. Graduation
6. Error

1. Picture 1
2. Error
3. Picture 2
4. Picture 3
5. Picture 4
6. Picture 5

1. Wedding
2. Birthday
3. Christmas
4. Easter
5. Graduation
6. Error

1. Picture 1
2. Error
3. Picture 2
4. Picture 3
5. Picture 4
6. Picture 5

1. Wedding
2. Birthday
3. Christmas
4. Easter
5. Graduation
6. Error

1. Picture 1
2. Error
3. Picture 2
4. Picture 3
5. Picture 4
6. Picture 5

1. Wedding
2. Birthday
3. Christmas
4. Easter
5. Graduation
6. Error

1. Picture 1
2. Error
3. Picture 2
4. Picture 3
5. Picture 4
6. Picture 5

1. Music
2. TV
3. Read
4. Photo
5. Radio
6. Other

1. Error
2. Trips I
3. Trips II
4. Events
5. Family & friends
6. Pets

1. Album 1
2. Album 2
3. Album 3
4. Album 4
5. Album 5
6. Error

1. Picture 1
2. Error
3. Picture 2
4. Picture 3
5. Picture 4
6. Picture 5

1. Album 1
2. Album 2
3. Album 3
4. Album 4
5. Album 5
6. Error

1. Picture 1
2. Error
3. Picture 2
4. Picture 3
5. Picture 4
6. Picture 5

1. Album 1
2. Album 2
3. Album 3
4. Album 4
5. Album 5
6. Error

1. Picture 1
2. Error
3. Picture 2
4. Picture 3
5. Picture 4
6. Picture 5

1. Album 1
2. Album 2
3. Album 3
4. Album 4
5. Album 5
6. Error

1. Picture 1
2. Error
3. Picture 2
4. Picture 3
5. Picture 4
6. Picture 5

1. Album 1
2. Album 2
3. Album 3
4. Album 4
5. Album 5
6. Error

1. Picture 1
2. Error
3. Picture 2
4. Picture 3
5. Picture 4
6. Picture 5

1. Music
2. TV
3. Read
4. Photo
5. Radio
6. Other

1. Error
2. Trips I
3. Trips II
4. Events
5. Family & friends
6. Pets

1. Album 1
2. Album 2
3. Album 3
4. Album 4
5. Album 5
6. Error

1. Picture 1
2. Error
3. Picture 2
4. Picture 3
5. Picture 4
6. Picture 5

1. Album 1
2. Album 2
3. Album 3
4. Album 4
5. Album 5
6. Error

1. Picture 1
2. Error
3. Picture 2
4. Picture 3
5. Picture 4
6. Picture 5

1. Album 1
2. Album 2
3. Album 3
4. Album 4
5. Album 5
6. Error

1. Picture 1
2. Error
3. Picture 2
4. Picture 3
5. Picture 4
6. Picture 5

1. Album 1
2. Album 2
3. Album 3
4. Album 4
5. Album 5
6. Error

1. Picture 1
2. Error
3. Picture 2
4. Picture 3
5. Picture 4
6. Picture 5

1. Album 1
2. Album 2
3. Album 3
4. Album 4
5. Album 5
6. Error

1. Picture 1
2. Error
3. Picture 2
4. Picture 3
5. Picture 4
6. Picture 5

Radio

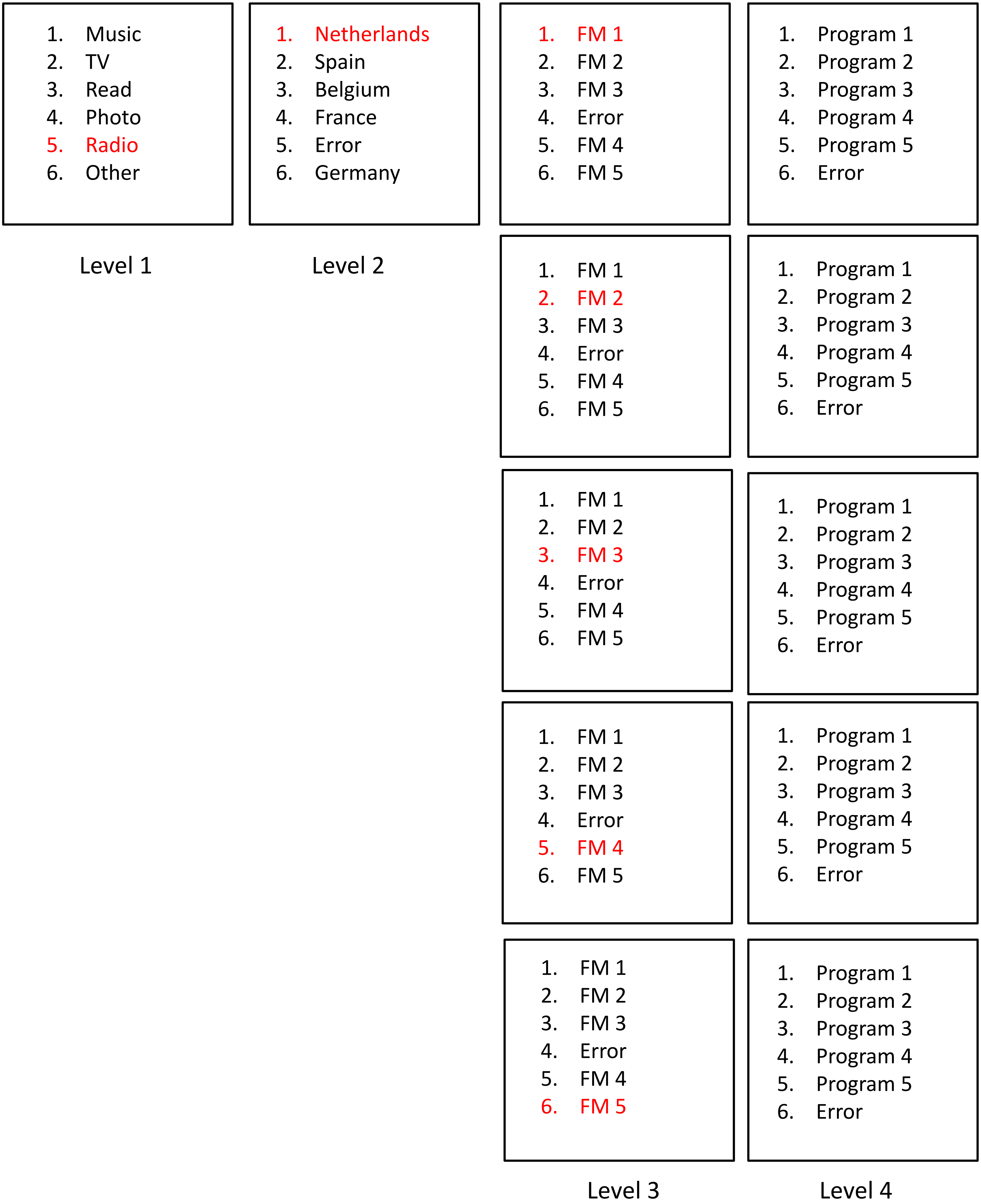

1. Music
2. TV
3. Read
4. Photo
5. Radio
6. Other
Level 1
1. Netherlands
2. Spain
3. Belgium
4. France
5. Error
6. Germany
Level 2
1. FM 1
2. FM 2
3. FM 3
4. Error
5. FM 4
6. FM 5
1. Program 1
2. Program 2
3. Program 3
4. Program 4
5. Program 5
6. Error
1. FM 1
2. FM 2
3. FM 3
4. Error
5. FM 4
6. FM 5
1. Program 1
2. Program 2
3. Program 3
4. Program 4
5. Program 5
6. Error
1. FM 1
2. FM 2
3. FM 3
4. Error
5. FM 4
6. FM 5
1. Program 1
2. Program 2
3. Program 3
4. Program 4
5. Program 5
6. Error
1. FM 1
2. FM 2
3. FM 3
4. Error
5. FM 4
6. FM 5
1. Program 1
2. Program 2
3. Program 3
4. Program 4
5. Program 5
6. Error
1. FM 1
2. FM 2
3. FM 3
4. Error
5. FM 4
6. FM 5
1. Program 1
2. Program 2
3. Program 3
4. Program 4
5. Program 5
6. Error
Level 3
Level 4

1. Music
2. TV
3. Read
4. Photo
5. Radio
6. Other

1. Netherlands
2. Spain
3. Belgium
4. France
5. Error
6. Germany

1. FM 1
2. FM 2
3. FM 3
4. Error
5. FM 4
6. FM 5

1. Program 1
2. Program 2
3. Program 3
4. Program 4
5. Program 5
6. Error

1. FM 1
2. FM 2
3. FM 3
4. Error
5. FM 4
6. FM 5

1. Program 1
2. Program 2
3. Program 3
4. Program 4
5. Program 5
6. Error

1. FM 1
2. FM 2
3. FM 3
4. Error
5. FM 4
6. FM 5

1. Program 1
2. Program 2
3. Program 3
4. Program 4
5. Program 5
6. Error

1. FM 1
2. FM 2
3. FM 3
4. Error
5. FM 4
6. FM 5

1. Program 1
2. Program 2
3. Program 3
4. Program 4
5. Program 5
6. Error

1. FM 1
2. FM 2
3. FM 3
4. Error
5. FM 4
6. FM 5

1. Program 1
2. Program 2
3. Program 3
4. Program 4
5. Program 5
6. Error

1. Music
2. TV
3. Read
4. Photo
5. Radio
6. Other

1. Netherlands
2. Spain
3. Belgium
4. France
5. Error
6. Germany

1. FM 1
2. FM 2
3. FM 3
4. Error
5. FM 4
6. FM 5

1. Program 1
2. Program 2
3. Program 3
4. Program 4
5. Program 5
6. Error

1. FM 1
2. FM 2
3. FM 3
4. Error
5. FM 4
6. FM 5

1. Program 1
2. Program 2
3. Program 3
4. Program 4
5. Program 5
6. Error

1. FM 1
2. FM 2
3. FM 3
4. Error
5. FM 4
6. FM 5

1. Program 1
2. Program 2
3. Program 3
4. Program 4
5. Program 5
6. Error

1. FM 1
2. FM 2
3. FM 3
4. Error
5. FM 4
6. FM 5

1. Program 1
2. Program 2
3. Program 3
4. Program 4
5. Program 5
6. Error

1. FM 1
2. FM 2
3. FM 3
4. Error
5. FM 4
6. FM 5

1. Program 1
2. Program 2
3. Program 3
4. Program 4
5. Program 5
6. Error

1. Music
2. TV
3. Read
4. Photo
5. Radio
6. Other

---

1. Netherlands
2. Spain
3. Belgium
4. France
5. Error
6. Germany

---

1. FM 1
2. FM 2
3. FM 3
4. Error
5. FM 4
6. FM 5

---

1. Program 1
2. Program 2
3. Program 3
4. Program 4
5. Program 5
6. Error

---

1. FM 1
2. FM 2
3. FM 3
4. Error
5. FM 4
6. FM 5

---

1. Program 1
2. Program 2
3. Program 3
4. Program 4
5. Program 5
6. Error

---

1. FM 1
2. FM 2
3. FM 3
4. Error
5. FM 4
6. FM 5

---

1. Program 1
2. Program 2
3. Program 3
4. Program 4
5. Program 5
6. Error

---

1. FM 1
2. FM 2
3. FM 3
4. Error
5. FM 4
6. FM 5

---

1. Program 1
2. Program 2
3. Program 3
4. Program 4
5. Program 5
6. Error

---

1. FM 1
2. FM 2
3. FM 3
4. Error
5. FM 4
6. FM 5

---

1. Program 1
2. Program 2
3. Program 3
4. Program 4
5. Program 5
6. Error

1. Music
2. TV
3. Read
4. Photo
5. Radio
6. Other

---

1. Netherlands
2. Spain
3. Belgium
4. France
5. Error
6. Germany

---

1. FM 1
2. FM 2
3. FM 3
4. Error
5. FM 4
6. FM 5

---

1. Program 1
2. Program 2
3. Program 3
4. Program 4
5. Program 5
6. Error

---

1. FM 1
2. FM 2
3. FM 3
4. Error
5. FM 4
6. FM 5

---

1. Program 1
2. Program 2
3. Program 3
4. Program 4
5. Program 5
6. Error

---

1. FM 1
2. FM 2
3. FM 3
4. Error
5. FM 4
6. FM 5

---

1. Program 1
2. Program 2
3. Program 3
4. Program 4
5. Program 5
6. Error

---

1. FM 1
2. FM 2
3. FM 3
4. Error
5. FM 4
6. FM 5

---

1. Program 1
2. Program 2
3. Program 3
4. Program 4
5. Program 5
6. Error

---

1. FM 1
2. FM 2
3. FM 3
4. Error
5. FM 4
6. FM 5

---

1. Program 1
2. Program 2
3. Program 3
4. Program 4
5. Program 5
6. Error

Other

1. Music
2. TV
3. Read
4. Photo
5. Radio
6. **Other**

Level 1

1. **Room control**
2. Error
3. Call
4. Admin. doc
5. Internet
6. Cube Settings

Level 2

1. **Window/Door**
2. Light
3. Error
4. Visits
5. Temperature
6. Bed

1. Error
2. Open window
3. Close window
4. Half open window
5. Close door
6. Open door

1. Window/Door
2. **Light**
3. Error
4. Visits
5. Temperature
6. Bed

1. Error
2. Close blinds
3. Open blinds
4. Switch on
5. Switch off
6. Dimmer

1. Window/Door
2. Light
3. Error
4. **Visits**
5. Temperature
6. Bed

1. Error
2. Allow now
3. Allow later
4. Not now
5. Not later
6. Not today

1. Window/Door
2. Light
3. Error
4. Visits
5. **Temperature**
6. Bed

1. Error
2. 20º
3. 22º
4. 18º
5. Higher
6. Lower

1. Window/Door
2. Light
3. Error
4. Visits
5. Temperature
6. **Bed**

1. Error
2. Move up
3. Move down
4. Full flat
5. Upright
6. Legs up

Level 3

Level 4

1. Music
2. TV
3. Read
4. Photo
5. Radio
6. Other

1. Room control
2. Error
3. Call
4. Admin. doc
5. Internet
6. Cube Settings

1. Doctor
2. Nurse
3. Error
4. Family
5. Friends
6. Care taker

1. Error
2. Doctor 1
3. Doctor 2
4. Doctor 3
5. Doctor 4
6. Doctor 5

1. Doctor
2. Nurse
3. Error
4. Family
5. Friends
6. Care taker

1. Error
2. Nurse 1
3. Nurse 2
4. Nurse 3
5. Nurse 4
6. Nurse 5

1. Doctor
2. Nurse
3. Error
4. Family
5. Friends
6. Care taker

1. Error
2. Mother
3. Father
4. Brother
5. Sister
6. Aunt

1. Doctor
2. Nurse
3. Error
4. Family
5. Friends
6. Care taker

1. Error
2. Friend 1
3. Friend 2
4. Friend 3
5. Friend 4
6. Friend 5

1. Doctor
2. Nurse
3. Error
4. Family
5. Friends
6. Care taker

1. Error
2. Caretaker 1
3. Caretaker 2
4. Caretaker 3
5. Caretaker 4
6. Caretaker 5

1. Music
2. TV
3. Read
4. Photo
5. Radio
6. Other

1. Room control
2. Error
3. Call
4. Admin. doc
5. Internet
6. Cube Settings

1. Financial
2. Health
3. Error
4. Insurance
5. Mortgage
6. Personal

1. Error
2. Sign doc
3. Read doc
4. Send doc
5. Edit doc
6. Save doc

1. Financial
2. Health
3. Error
4. Insurance
5. Mortgage
6. Personal

1. Error
2. Sign doc
3. Read doc
4. Send doc
5. Edit doc
6. Save doc

1. Financial
2. Health
3. Error
4. Insurance
5. Mortgage
6. Personal

1. Error
2. Sign doc
3. Read doc
4. Send doc
5. Edit doc
6. Save doc

1. Financial
2. Health
3. Error
4. Insurance
5. Mortgage
6. Personal

1. Error
2. Sign doc
3. Read doc
4. Send doc
5. Edit doc
6. Save doc

1. Financial
2. Health
3. Error
4. Insurance
5. Mortgage
6. Personal

1. Error
2. Sign doc
3. Read doc
4. Send doc
5. Edit doc
6. Save doc

1. Music
2. TV
3. Read
4. Photo
5. Radio
6. Other

1. Room control
2. Error
3. Call
4. Admin. doc
5. Internet
6. Cube Settings

1. Browse info
2. Online game
3. Error
4. Youtube
5. Visit museum
6. Google earth

1. Error
2. Keyword
3. Sentence
4. Image
5. News
6. Feeling lucky

1. Browse info
2. Online game
3. Error
4. Youtube
5. Visit museum
6. Google earth

1. Error
2. 1 player
3. 2 players
4. 3 players
5. 4 players
6. 5 players

1. Browse info
2. Online game
3. Error
4. Youtube
5. Visit museum
6. Google earth

1. Error
2. Funny videos
3. Concerts
4. Tutorials
5. Gaming
6. Speeches

1. Browse info
2. Online game
3. Error
4. Youtube
5. Virtual museum
6. Google earth

1. Error
2. New York
3. Chicago
4. Rome
5. London
6. Madrid

1. Browse info
2. Online game
3. Error
4. Youtube
5. Visit museum
6. Google earth

1. Error
2. Zoom in
3. Zoom out
4. Move East
5. Move North
6. Move South

1. Music
2. TV
3. Read
4. Photo
5. Radio
6. Other

1. Room control
2. Error
3. Call
4. Admin. doc
5. Internet
6. Cube Settings

1. Size
2. Color
3. Error
4. Font type
5. Rotation speed
6. Encoding time

1. Error
2. Very small
3. Small
4. Medium
5. Big
6. Very big

1. Size
2. Color
3. Error
4. Font type
5. Rotation speed
6. Encoding time

1. Error
2. Colormap A
3. Colormap B
4. Colormap C
5. Colormap D
6. Colormap E

1. Size
2. Color
3. Error
4. Font type
5. Rotation speed
6. Encoding time

1. Error
2. Arial
3. Helvetica
4. Corbel
5. Calibri
6. Comic Sana

1. Size
2. Color
3. Error
4. Font type
5. Rotation speed
6. Encoding time

1. Error
2. Very slow
3. Slow
4. Medium
5. Fast
6. Very fast

1. Size
2. Color
3. Error
4. Font type
5. Rotation speed
6. Encoding time

1. Error
2. 2s
3. 3s
4. 6s
5. 7s
6. 8s
